# Genetic associations of adult height with risk of cardioembolic and other subtypes of ischemic stroke: A mendelian randomization study in multiple ancestries

**DOI:** 10.1371/journal.pmed.1003967

**Published:** 2022-04-22

**Authors:** Andrew B. Linden, Robert Clarke, Imen Hammami, Jemma C. Hopewell, Yu Guo, William N. Whiteley, Kuang Lin, Iain Turnbull, Yiping Chen, Canqing Yu, Jun Lv, Alison Offer, Derrick Bennett, Robin G. Walters, Liming Li, Zhengming Chen, Sarah Parish, for the China Kadoorie Biobank Collaborative Group

**Affiliations:** 1 Clinical Trial Service Unit and Epidemiological Studies Unit, Nuffield Department of Population Health, University of Oxford, Oxford, United Kingdom; 2 Chinese Academy of Medical Sciences, Beijing, China; 3 Centre for Clinical Brain Sciences, University of Edinburgh, Edinburgh, United Kingdom; 4 MRC Population Health Research Unit, Nuffield Department of Population Health, University of Oxford, Oxford, United Kingdom; 5 Peking University Health Science Center, Beijing, China; Columbia University, UNITED STATES

## Abstract

**Background:**

Taller adult height is associated with lower risks of ischemic heart disease in mendelian randomization (MR) studies, but little is known about the causal relevance of height for different subtypes of ischemic stroke. The present study examined the causal relevance of height for different subtypes of ischemic stroke.

**Methods and findings:**

Height-associated genetic variants (up to 2,337) from previous genome-wide association studies (GWASs) were used to construct genetic instruments in different ancestral populations. Two-sample MR approaches were used to examine the associations of genetically determined height with ischemic stroke and its subtypes (cardioembolic stroke, large-artery stroke, and small-vessel stroke) in multiple ancestries (the MEGASTROKE consortium, which included genome-wide studies of stroke and stroke subtypes: 60,341 ischemic stroke cases) supported by additional cases in individuals of white British ancestry (UK Biobank [UKB]: 4,055 cases) and Chinese ancestry (China Kadoorie Biobank [CKB]: 10,297 cases). The associations of genetically determined height with established cardiovascular and other risk factors were examined in 336,750 participants from UKB and 58,277 participants from CKB. In MEGASTROKE, genetically determined height was associated with a 4% lower risk (odds ratio [OR] 0.96; 95% confidence interval [CI] 0.94, 0.99; *p* = 0.007) of ischemic stroke per 1 standard deviation (SD) taller height, but this masked a much stronger positive association of height with cardioembolic stroke (13% higher risk, OR 1.13 [95% CI 1.07, 1.19], *p* < 0.001) and stronger inverse associations with large-artery stroke (11% lower risk, OR 0.89 [0.84, 0.95], *p* < 0.001) and small-vessel stroke (13% lower risk, OR 0.87 [0.83, 0.92], *p* < 0.001). The findings in both UKB and CKB were directionally concordant with those observed in MEGASTROKE, but did not reach statistical significance: For presumed cardioembolic stroke, the ORs were 1.08 (95% CI 0.86, 1.35; *p* = 0.53) in UKB and 1.20 (0.77, 1.85; *p* = 0.43) in CKB; for other subtypes of ischemic stroke in UKB, the OR was 0.97 (95% CI 0.90, 1.05; *p* = 0.49); and for other nonlacunar stroke and lacunar stroke in CKB, the ORs were 0.89 (0.80, 1.00; *p* = 0.06) and 0.99 (0.88, 1.12; *p* = 0.85), respectively. In addition, genetically determined height was also positively associated with atrial fibrillation (available only in UKB), and with lean body mass and lung function, and inversely associated with low-density lipoprotein (LDL) cholesterol in both British and Chinese ancestries. Limitations of this study include potential bias from assortative mating or pleiotropic effects of genetic variants and incomplete generalizability of genetic instruments to different populations.

**Conclusions:**

The findings provide support for a causal association of taller adult height with higher risk of cardioembolic stroke and lower risk of other ischemic stroke subtypes in diverse ancestries. Further research is needed to understand the shared biological and physical pathways underlying the associations between height and stroke risks, which could identify potential targets for treatments to prevent stroke.

## Abstract 2

In a Mendelian randomization study, Andrew B. Linden and colleagues study the relationship between height and risk of stroke subtypes among individuals from the MEGASTROKE consortium, China Kadoorie Biobank, and UK Biobank.

## Author summary

**Why was this study done?:**

- Taller people have lower risks of ischemic stroke and heart disease, but higher risks of atrial fibrillation. However, little is known about the effects of height on the risks of different subtypes of ischemic stroke (cardioembolic stroke, large-artery stroke, and small-vessel stroke).
- Understanding the shared biological and physical pathways underlying the associations between height and stroke risks could identify potential targets for treatments to prevent stroke.
- Mean height and the rates of different stroke subtypes vary considerably across different income and ancestry populations, and, therefore, investigation across diverse ancestries is important.

**What did the researchers do and find?:**

- We used a mendelian randomization (MR) approach to study the association between genetic variants for height and risk of ischemic stroke subtypes in populations with different ancestries.
- Genetic variants associated with taller height were associated with higher risks of cardioembolic stroke and lower risks of large-artery and small-vessel stroke.
- The findings were consistent across populations of different genetic ancestries and use of different analytical methods.

**What do these findings mean?:**

- The findings support a causal association of taller adult height with higher risks of atrial fibrillation and cardioembolic stroke and lower risks of other ischemic stroke subtypes.
- Further research is needed to clarify the biological and physical pathways underlying the associations of height with ischemic stroke subtypes, which could identify novel targets for treatments to prevent stroke.

## Introduction

Taller people have lower risks of atherosclerotic diseases, ischemic stroke, and heart disease, but higher risks of atrial fibrillation and venous thromboembolism [1–3]. The associations of height with ischemic stroke subtypes have not been reported, but it would be of interest to know whether these vary between atherosclerotic and cardioembolic stroke subtypes. In observational studies, any such associations could reflect confounding by socioeconomic status or other known or unknown correlates of height that are risk factors for cardiovascular diseases. Alternatively, the associations could be causal and could possibly be mediated through physical effects of height on body structure (including lean body mass or lung function) [4–7].

Increasingly, mendelian randomization (MR) analyses have been used to assess the causal relevance of risk factors for diseases by using genetic variants associated with risk factors of interest as instrumental variables [8]. The allocation of genetic variants to gametes (and hence offspring) is determined randomly at meiosis. Therefore, the random distribution of variants for a trait, such as height, between individuals can be used to minimize the effects of confounding by risk factors and provide support for the causal relevance of the trait for disease outcomes. Previous MR studies have reported that genetically determined differences in adult height were inversely associated with ischemic heart disease [4] and hypertension [2], but positively associated with atrial fibrillation [2,3], venous thromboembolism [2], and vasculitis [2]. However, the associations of genetically determined height with ischemic stroke and ischemic stroke subtypes have not been reliably established as previous studies have focused analyses on total stroke rather than on individual stroke pathological types and their main subtypes [2,9].

The present study examined the observational and genetic associations (using MR approaches) of height with (i) ischemic stroke and subtypes of ischemic stroke in the MEGASTROKE consortium (an international collaboration on the genetics of stroke) and in 2 large prospective studies conducted in the United Kingdom and China [10,11]; and (ii) established cardiovascular risk factors and anthropometric traits in the 2 large prospective studies.

## Methods

This study is reported using the Strengthening the Reporting of Observational Studies in Epidemiology using Mendelian Randomization (STROBE-MR) [12] guideline (S1 Checklist). The study did not have a prospective protocol or published analysis plan. Analyses were planned prior to study initiation, but some were subsequently revised to reflect availability of new data or in response to reviewer comments (S1 Methods).

### MEGASTROKE

MEGASTROKE consortium data included 29 genome-wide studies of stroke and stroke subtypes [13]. Ischemic stroke cases were defined using standard diagnostic criteria based on clinical and imaging findings and were further classified into subtypes using the Trial of ORG 10 172 in Acute Stroke Treatment (TOAST) criteria [13,14]. Analyses were conducted using meta-analyzed, heterogeneity-filtered summary results from multiple ancestries (60,341 ischemic stroke cases—including 9,006 cardioembolic stroke, 6,688 large-artery atherosclerotic stroke, and 11,710 small-vessel stroke subtypes—and up to 454,450 controls) and separately for the subset of Europeans (34,217 ischemic stroke cases) [13]. Summary results for separate non-European ancestries were not made available by the consortium.

### UK Biobank

The UK Biobank (UKB) is a prospective study of 502,506 men and women, aged 40 to 69 years living in the UK, who were enrolled between 2006 and 2010 [10,15]. All participants provided written informed consent to participate in a study defined by a protocol approved by the North West Multi-centre Research Ethics Committee on May 10, 2016 (reference: 16/NW/0274). Details of the study methods and baseline characteristics have been previously reported (S2 Methods) [10,15]. Participants were followed up for a mean of 8 years through linkage to death registries and hospital admission records. Criteria for diagnosis of ischemic stroke cases (ICD-10: I63) were prespecified and included both cases recorded prior to enrollment and incident cases recorded during follow-up (S2 Methods). Ischemic stroke cases with a history of atrial fibrillation, based on either a self-reported diagnosis at baseline or an admission to hospital (ICD-10: I48) prior to onset of the stroke, were classified as having presumed cardioembolic stroke (S2 Methods). The remaining noncardioembolic ischemic stroke cases were classified as other subtypes of ischemic stroke. Genotyping using Affymetrix arrays with imputation into multiple reference panels was available for 483,420 participants passing quality control (S2 Methods). After exclusions for non-white British ancestry (*n* = 78,674) and relatedness (*n* = 67,201; kinship coefficient ≥0.125), a total of 336,750 UKB participants were included in the present genetic analyses (S1 Fig).

### China Kadoorie Biobank

The China Kadoorie Biobank (CKB) is a prospective study of 513,214 men and women, aged 30 to 79 years, who were enrolled from 10 (5 urban and 5 rural) geographically defined regions of China between 2004 and 2008 [11]. All participants provided written informed consent to participate in a study defined by a protocol that was approved by the Oxford Tropical Research Ethics Committee on February 3, 2005 (reference: 025–04) and by the Ethics Review Committee of the Chinese Center for Disease Control and Prevention on July 8, 2004 (approval notice: 005/2004). Details of the study methods and baseline characteristics have been previously reported (S3 Methods) [11]. Compared to participants in UKB, those in CKB were on average 5 years younger (mean age 51.6 [standard deviation (SD) 10.6] versus 56.4 [8.1] years) and were less highly educated (S1 Table). Participants were followed up for a mean of 10 years through linkages to death and stroke registries and health insurance claims records. Adjudication of stroke was undertaken by review of clinical findings from medical records and brain imaging reports (available for >92% of stroke cases with retrieved records) by specialist clinicians using a defined protocol (S3 Methods). Presumed cardioembolic strokes were identified from confirmed ischemic stroke cases based on the Causative Classification System criteria [16]. Other confirmed ischemic stroke cases were further classified by brain infarct size into lacunar and other nonlacunar stroke subtypes. Data on atrial fibrillation were not systematically recorded at baseline or during follow-up in CKB, but electrocardiographic evidence of atrial fibrillation and other major and minor sources of cardioembolism were recorded by adjudicating physicians. Genotyping using Affymetrix arrays with imputation into the 1000 Genomes reference panel (S3 Methods) was available for 100,706 participants passing quality control, comprising a sample of 76,020 participants selected to be representative of the CKB population [17] and an additional 24,686 selected for nested case–control studies of incident cardiovascular or respiratory disease (S3 Methods). After relatedness exclusions (*n* = 28,233; kinship coefficient >0.05), the present genetic analyses involved 58,277 CKB participants (53,346 from the population-based subset and 4,931 additional ischemic stroke cases included only in analyses of ischemic stroke outcomes; S2 Fig).

### Height

Participants in CKB were on average shorter (10 cm in men, 8 cm in women; S1 Table) than those in UKB and the SDs of directly measured height in UKB and CKB, respectively, were 6.8 cm and 6.5 cm in men, and 6.3 cm and 6.0 cm in women. Separately in UKB and CKB, following the methodology used in the Genetic Investigation of Anthropometric Traits (GIANT) consortium, a measured height phenotype was constructed: Within strata by sex (and by region in CKB), directly measured height (S2 and S3 Methods) was adjusted for age and age^2^, and the residuals were transformed using an inverse normal transformation, yielding a measured height phenotype in study and sex-specific SD units. This transformed height phenotype (referred to as “height” or “measured height”) was used for all analyses (unless “directly measured” is explicitly stated).

### Blood pressure, blood lipids, and other anthropometric traits

Systolic and diastolic blood pressure were measured using standard instruments and protocols. Blood lipids (low-density lipoprotein [LDL] cholesterol, high-density lipoprotein [HDL] cholesterol, triglycerides, and apolipoprotein B) [17] were available in a subset of CKB participants (S2 Fig). Fat body mass was estimated as weight multiplied by percentage body fat measured by bio-impedance (S2 and S3 Methods). Lean body mass was estimated as weight minus fat body mass. Lung function measures (forced vital capacity [FVC] and forced expiratory volume in 1 second [FEV1]) were restricted to those with reliable values (S2 and S3 Methods, S2 Fig). Compared with UKB participants, those in CKB had lower mean levels of systolic blood pressure (7.3 mm Hg), diastolic blood pressure (4.7 mm Hg), LDL cholesterol (1.2 mmol/L), HDL cholesterol (0.3 mmol/L), apolipoprotein B (0.2 g/L), body mass index (BMI; 4 kg/m^2^ in men and 3 kg/m^2^ in women), and lean body mass (14 kg in men and 7 kg in women), but higher mean levels of triglycerides (0.3 mmol/L; S1 Table).

### Instruments for genetically determined height

Genetic instruments for a 2-sample MR approach were constructed separately for MEGASTROKE, UKB, and CKB, due to differences in ancestry and overlap in participants in genome-wide association studies (GWASs) of height. For MEGASTROKE, height-associated single nucleotide polymorphisms (SNPs) from the GIANT GWAS report in 2018 [18] (which also included data from the whole of UKB) were used for both multiple and European ancestry analyses (S2 Table). For UKB, the genetic instrument was constructed from height-associated SNPs obtained from an earlier (2014) GIANT study that was independent of UKB [19]. For CKB, both the European ancestry–based GIANT GWAS (2018) [18] and a smaller GWAS from Biobank Japan [20], involving participants of East Asian ancestry, were used to optimize the genetic instrument for height by benefitting from a larger discovery population and a more proximal genetic ancestry [21,22].

The SNPs selected from these GWAS studies (together with their published single-variant effect sizes on height) were those associated with height at genome-wide significance and also available in MEGASTROKE, UKB, or CKB (S4 Methods). The SNPs from each GWAS were linkage disequilibrium (LD) pruned (r^2^ < 0.05) using LD estimates from UKB for GIANT and from CKB for Biobank Japan (i.e., where r^2^ between SNPs was ≥0.05, the SNP with the lowest *p*-value for association with height in the GWAS was retained). Palindromic SNPs were validated by comparing allele frequencies for individual participant data (UKB and CKB). For MEGASTROKE, palindromic SNPs were replaced with high LD proxies (r^2^ > 0.9).

After LD pruning, 641 height-associated SNPs from GIANT were available for analysis in UKB (S4 Methods). Likewise, 2,337 height-associated SNPs from GIANT (European ancestry) and 517 SNPs from Biobank Japan (East Asian ancestry) were available for analysis in CKB. In MEGASTROKE, after LD pruning (at *p* < 0.05) and replacing palindromic SNPs with proxies, the number of height-associated SNPs from GIANT remaining for analysis available in each of the multiple ancestry summary data sets was 2,265 for ischemic stroke, 2,270 for cardioembolic and large-artery stroke, and 2,084 for small-vessel stroke. The SNPs used in MEGASTROKE, UKB, and CKB are listed in S1–S3 Data Tables.

For UKB and CKB, genetic risk scores for each individual were constructed as the sum of the number of each height-associated effect alleles weighted by their published single-variant effect sizes on height (S4 Methods, S2 and S3 Data Tables). For CKB, the genetic risk score was the simple average of weighted genetic risk scores constructed from 2,337 GIANT (2018) [18] and 517 Biobank Japan [20] height-associated SNPs (other percentages of the 2 genetic risk scores, including either score alone, were assessed in sensitivity analyses but had less explanatory power; S3 Table). The effects of SNPs on height in UKB and CKB estimated separately for each SNP using linear regression adjusted for age, age^2^, sex, region (in CKB only), genomic principal components (40 in UKB and 14 in CKB), and genotyping array type were also compared with the published effect sizes on height.

**Fig 1:**
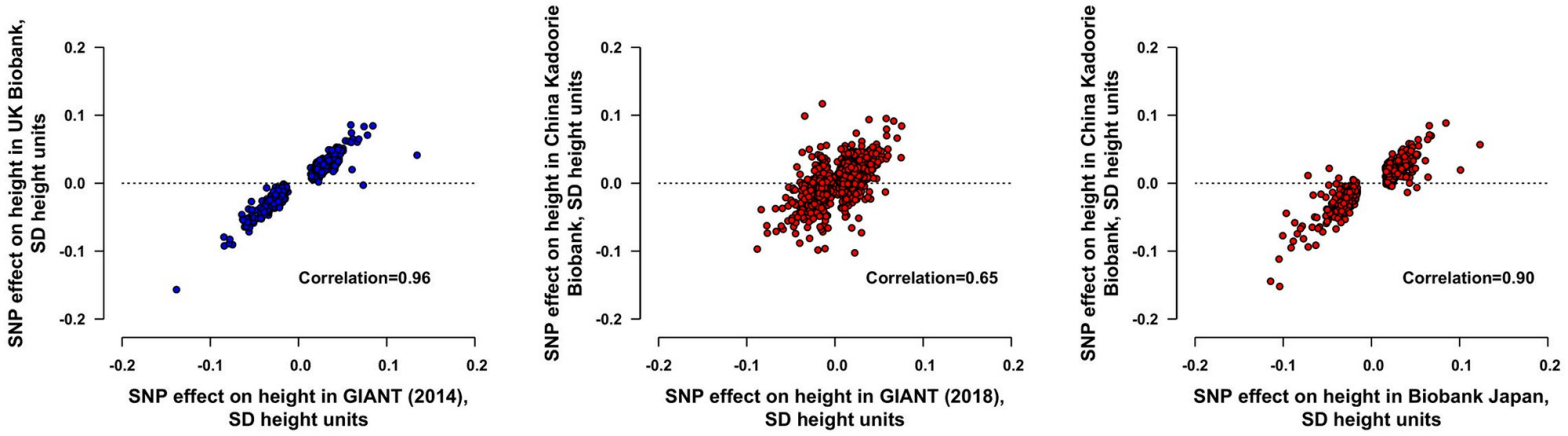
Effects of height-associated SNPs on height in UKB and CKB. For UKB (336,750 participants), the effects on height were estimated for 641 SNPs from GIANT (2014) [19]. For CKB (53,346 participants), the effects on height were estimated for 2,189/2,337 SNPs from GIANT (2018) [18] and 499/517 SNPs from Biobank Japan [20] with minor allele frequency ≥0.005 in CKB. The effect sizes on height were adjusted for age, age^2^, sex, region (in CKB only), genomic principal components, and genotyping array type. SNPs with minor allele frequencies of <0.005 were not shown. In UKB, the genetic risk score explained 17.0% of the variance of height and, in CKB, the genetic risk scores from GIANT (2018) [18], Biobank Japan [20], and the average genetic risk score, respectively, explained 11.4%, 11.0%, and 15.2% of the variance of height. CKB, China Kadoorie Biobank; GIANT, Genetic Investigation of Anthropometric Traits; SD, standard deviation; SNP, single nucleotide polymorphism; UKB, UK Biobank.

The genetic risk score in UKB explained 17.0% of the variance of height (S4 Methods, S3 Table) and the effect sizes of the SNPs in UKB were highly correlated with the effect sizes in the source GWAS [19] (r = 0.96; Fig 1). In CKB, the genetic risk scores from GIANT, Biobank Japan, and the average genetic risk score, respectively, explained 11.4%, 11.0%, and 15.2% of the variance of height (S3 Table). SNP effect sizes in CKB were less strongly correlated with effect sizes in GIANT (r = 0.65) [18], but were more strongly correlated with effect sizes in Biobank Japan (r = 0.90, respectively; Fig 1). One unit of the respective genetic risk score was associated with 0.91 SD of measured height in UKB and 1.05 SD in CKB.

### Genetic analyses

Since only GWAS summary results on stroke were available from MEGASTROKE [13] (and not individual participant data), causal effects were estimated by inverse-variance–weighted random-effects SNP-level meta-analysis [23] (S5 Methods, S3 and S4 Figs, S4 Data Table). For UKB and CKB, individual participant data were used to construct genetic risks scores for each individual, and the ratio method for single instruments was applied to estimate the genetically instrumented causal effects on outcomes per 1 SD of measured height. When using the ratio method, the second order variance term that is formally used in an instrumental variable estimate was ignored because the contribution from this term would be negligible given the strength (large F-statistics) of the instruments [23,24]. Specifically, logistic regression was used to assess associations of each genetic risk score with the stroke outcomes (after adjustment for age, age^2^, sex, region in CKB, genomic principal components, and genotyping array type). Subsequently, the coefficients from these regressions were divided by the regression coefficient of measured height on the genetic risk score (0.91 SD of measured height in UKB and 1.05 SD in CKB) to estimate the causal effects [23]. The genetic instruments used in the different populations were all strongly associated with height (F-statistic of 69,096 for UKB and 9,589 for CKB and an average F-statistic of 109 per genetic variant in MEGASTROKE). All effects presented as associations of genetically determined height are the instrumented effects per 1 SD higher measured level of height (S3 Fig).

To investigate the potential for factors to contribute to pleiotropy, cross-sectional associations of genetically determined height with established cardiovascular risk factors, and anthropometric traits were assessed in UKB and CKB using linear or logistic regression as appropriate, with adjustment for age, sex, region in CKB, genomic principal components, and genotyping array type. For these cross-sectional associations, anthropometric traits and lung function were standardized (by dividing by their SD within each sex) in the UKB and CKB populations. The ratio method was then applied to regression results and, as for the disease outcomes, the genetically instrumented effects presented. As t-statistics closely approximate z-statistics in large samples, they are referred to as z-statistics in this report. These were used to assess the strength and direction of the associations of height with cardiovascular and anthropometric factors to permit comparisons of z-statistics up to about ±500, which is beyond the convenient ranges for *p*-values (z-statistics of ±1.96 and of ±37 correspond to 2p = 0.05 and 2p ≈ 1 × 10^−300^, respectively).

### Sensitivity analyses

As MR inference relies on various assumptions (including instrumental variable assumptions) [24], additional sensitivity analyses in MEGASTROKE included weighted median analyses, MR–Egger analyses to assess any possible pleiotropic effects of height on other factors, and Mendelian Randomization Pleiotropy RESidual Sum and Outlier (MR–PRESSO) analyses to correct for pleiotropy, if any, by removal of outliers (S6 Methods) [25]. As there is some overlap of the populations in MEGASTROKE with those in GIANT (2018) [18] (S6 Methods) but not with UKB, the sensitivity analyses were repeated using effect sizes on height estimated in UKB. A further sensitivity analyses excluded SNPs that were associated at *p* < 0.001 in the large pan-ancestry UKB GWAS analyses [26] with age at completion of education, diabetes, atrial fibrillation, hypertension, systolic blood pressure, diastolic blood pressure, LDL cholesterol, HDL cholesterol, triglycerides, or apolipoprotein B (S6 Methods). An additional sensitivity analysis in MEGASTROKE used more stringent pruning criteria (r^2^ < 0.001) for SNP inclusion to provide greater comparability with recent literature. In CKB, the analyses of genetically determined height with ischemic stroke subtypes were repeated using separate genetic instruments constructed from GIANT (2018) [18] SNPs and from Biobank Japan [20] SNPs.

### Observational analyses

Observational analyses were restricted to participants with no prior history of ischemic heart disease or stroke in UKB (S1 Fig) and CKB (S2 Fig, S7 Methods). Hazard ratios (HRs) for the associations of measured height (grouped and as a linear term) with incident ischemic stroke and ischemic stroke subtypes postrecruitment were estimated by Cox regressions stratified by age at risk (in 5-year groups), sex, and region (10 regions in CKB), with adjustment for possible baseline confounders (S7 Methods). Cross-sectional associations of measured height with cardiovascular and anthropometric factors at baseline were assessed using linear or logistic regression as appropriate and adjusted for age (in 5-year groups), sex, year of birth, and region in CKB. All statistical analyses were conducted in SAS (version 9.4) and R (version 3.3.3) and are available upon request.

## Results

**Fig 2:**
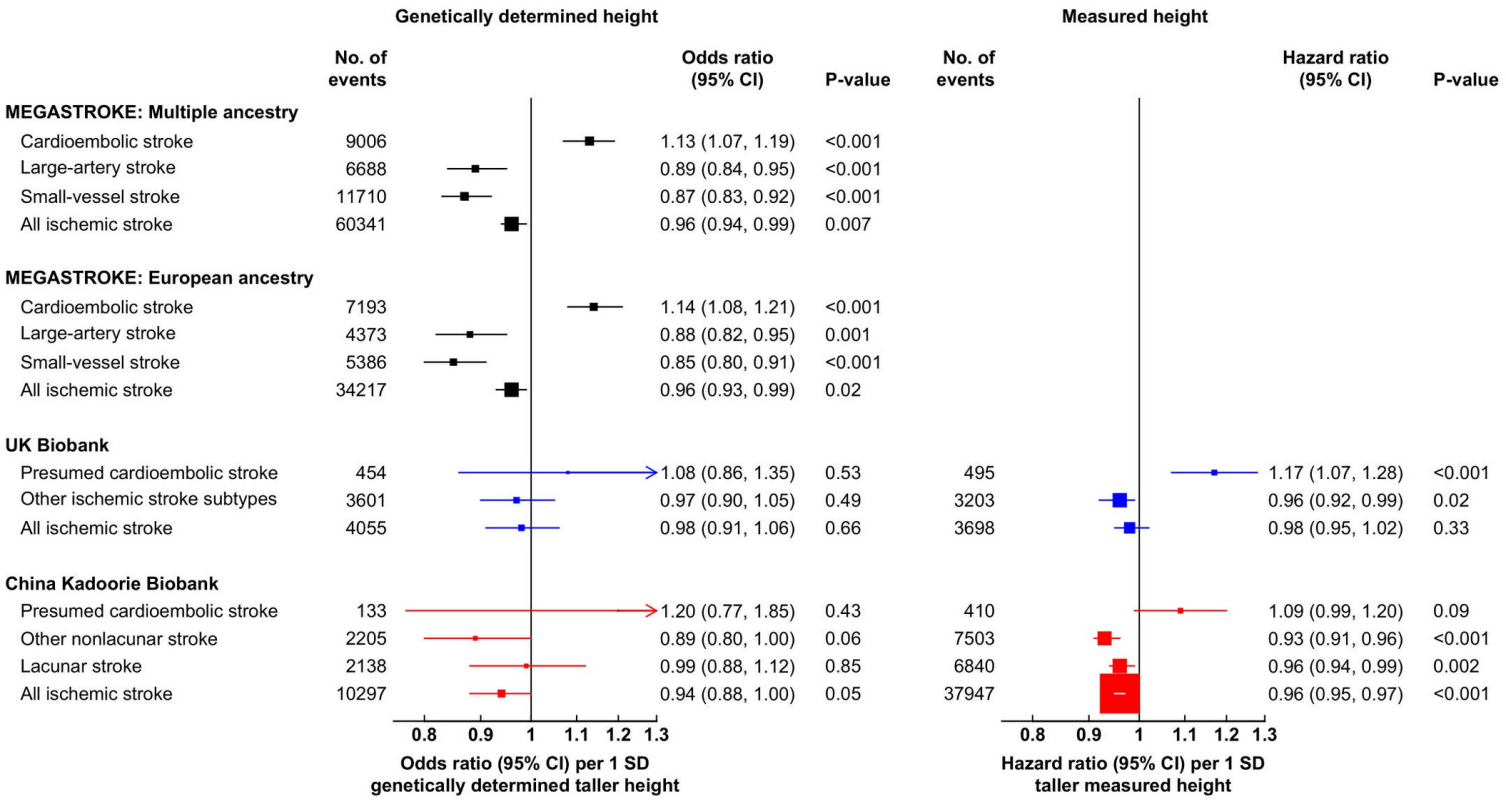
Associations of measured and genetically determined height with ischemic stroke and its subtypes in MEGASTROKE, UKB, and CKB. The numbers of events reported for MEGASTROKE were the maximum number of cases available in the genetic summary data. In MEGASTROKE and CKB, “All ischemic stroke” includes additional unsubtyped ischemic strokes. For UKB and CKB, respectively, the SDs of directly measured height were 6.8 cm versus 6.5 cm for men and 6.3 cm versus 6.0 cm for women. Genetic associations in UKB and CKB were adjusted for age, age^2^, sex, region (in CKB only), genomic principal components, and genotyping array type, and observational associations were stratified by age at risk (in 5-year groups), sex, and region (in CKB only) and adjusted for additional potential confounders (S6 Methods). CI, confidence interval; CKB, China Kadoorie Biobank; SD, standard deviation; UKB, UK Biobank.

Genetically determined height was inversely associated with ischemic stroke in MEGASTROKE in both multiple ancestries (odds ratio [OR]: 0.96; 95% confidence interval [CI]: 0.94, 0.99; *p* = 0.007) per 1 SD taller height, *n* = 60,341 cases) and the European ancestry subset (0.96 [0.93, 0.99]; *p* = 0.02; *n* = 34,217; Fig 2). The genetic associations with ischemic stroke in UKB (OR: 0.98 [95% CI 0.91, 1.06]; *p* = 0.66; *n* = 4,055) and CKB (0.94 [0.88, 1.00]; *p* = 0.05; *n* = 10,297) were also consistent with the results in MEGASTROKE (Fig 2). However, the results for overall ischemic stroke masked directionally opposing associations with different subtypes of ischemic stroke.

In MEGASTROKE, genetically determined height was positively associated with cardioembolic stroke (OR per 1 SD taller height: 1.13 [95% CI 1.07, 1.19]; *p* < 0.001; *n* = 9,006), but was inversely associated with large-artery stroke (0.89 [0.84, 0.95]; *p* < 0.001; *n* = 6,688) and small-vessel stroke (0.87 [0.83, 0.92]; *p* < 0.001; *n* = 11,710) in multiple ancestries and were similar in the European ancestry subset (Fig 2). The findings in both UKB and CKB were directionally concordant with the associations observed in MEGASTROKE, but did not reach statistical significance: For presumed cardioembolic stroke, the ORs were 1.08 (95% CI 0.86, 1.35; *p* = 0.53; *n* = 454 cases) in UKB and 1.20 (0.77, 1.85; *p* = 0.43; *n* = 133 cases) in CKB; for other subtypes of ischemic stroke, the corresponding ORs were 0.97 (95% CI 0.90, 1.05; *p* = 0.49; *n* = 3,601) in UKB, while in CKB, they were 0.89 (0.80, 1.00; *p* = 0.06; *n* = 2,205) for other nonlacunar stroke and 0.99 (0.88, 1.12; *p* = 0.85; *n* = 2,138) for lacunar stroke (Fig 2, S4 Table).

Sensitivity analyses in MEGASTROKE also demonstrated reliable concordant estimates irrespective of the methodology used for estimation, which included weighted median method, MR–Egger, and MR–PRESSO (S5 Table). Importantly, there was no evidence of directional pleiotropy for ischemic stroke or its subtypes (*p* > 0.08 for nonzero MR–Egger intercepts). The MR–PRESSO analyses identified only a few outlying SNPs (*n* ≤ 4), and their exclusion had no impact on the causal estimates. MR results remained similar when a restricted genetic instrument was used that consisted of the 1,515 (67%) of SNPs not associated at *p* < 0.001 with potentially pleiotropic risk factors for stroke (S6 Table). There was no evidence of bias due to sample overlap as the causal estimates based on UKB effect sizes on height were largely unchanged. In addition, the application of a stricter level of LD pruning (r^2^ < 0.001) had little impact on the causal estimates (S6 Table). In CKB, sensitivity analyses of the component genetic instruments for height yielded similar results to the combined instrument in the main analyses (S7 Table).

**Fig 3:**
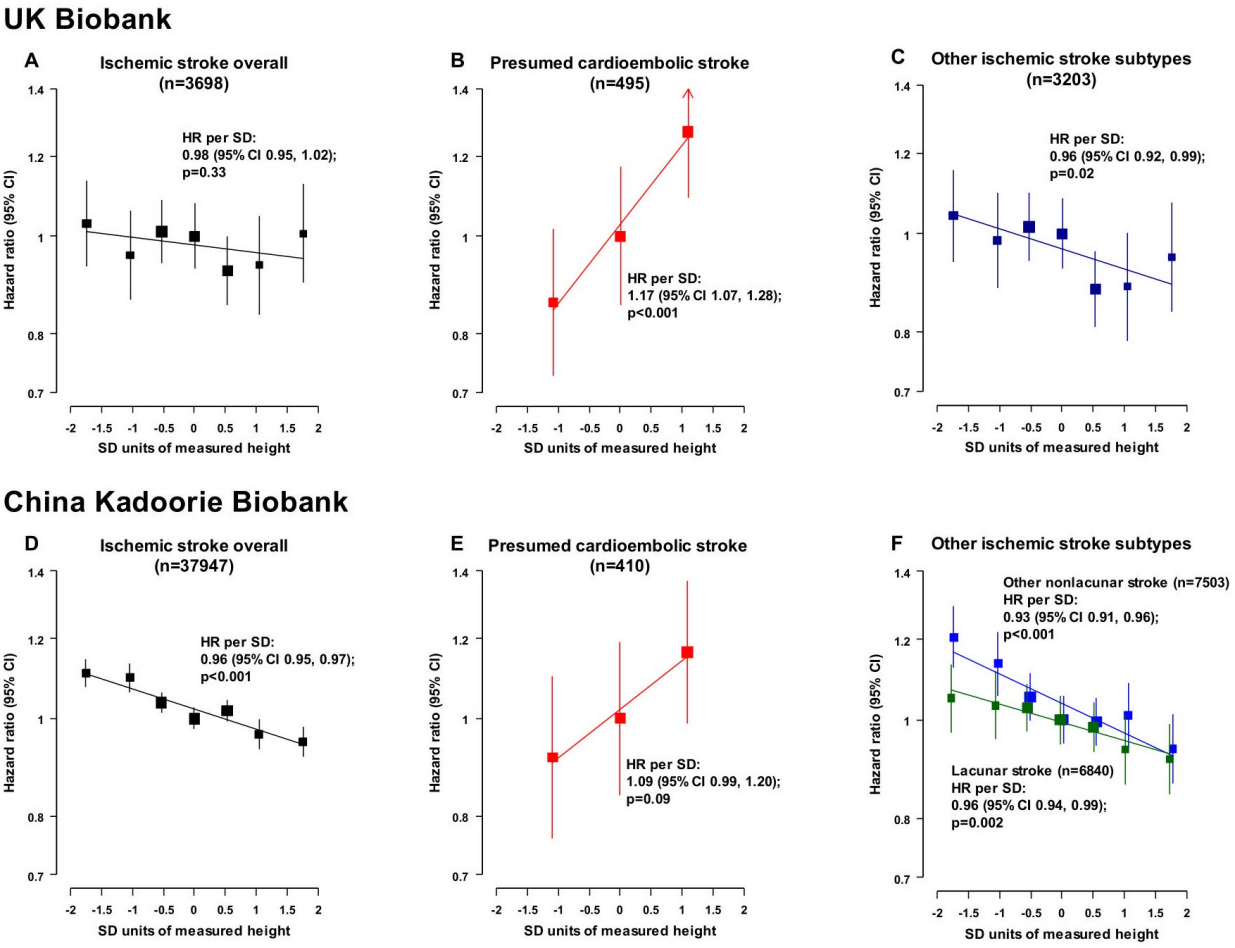
Associations of measured height with ischemic stroke and its subtypes in UKB and CKB. In UKB, the category “Other ischemic stroke subtypes” includes all ischemic strokes not classified as “Presumed cardioembolic stroke,” whereas in CKB, the category includes all subtyped ischemic strokes not classified as “Presumed cardioembolic stroke.” For UKB (482,928 participants) and CKB (490,067 participants), respectively, the SDs of directly measured height were 6.8 cm versus 6.5 cm for men and 6.3 cm versus 6.0 cm for women. HRs were stratified by age at risk (in 5-year groups), sex, and region (in CKB only) and adjusted for additional potential confounders (S6 Methods). Tenths of measured height were used to examine the shape of the associations of height with ischemic stroke subtypes, except for presumed cardioembolic stroke where thirds were used due to the lower number of cases. When tenths of height were plotted, consecutive pairs of the middle 6 tenths were combined (to give 7 groups). HRs were presented as floating absolute risks relative to the middle height category (whereby standard errors were assigned approximately independently to each category to avoid restricting comparisons to any arbitrary reference groups). CI, confidence interval; CKB, China Kadoorie Biobank; HR, hazard ratio; SD, standard deviation; UKB, UK Biobank.

Taller measured height was inversely and log-linearly associated with risk of ischemic stroke in both UKB (HR per 1 SD taller measured height: 0.98 [95% CI 0.95, 1.02]; *p* = 0.33; *n* = 3,698) and CKB (0.96 [0.95, 0.97]; *p* < 0.001; *n* = 37,947), although the association was not statistically significant in UKB (Fig 3). The associations of measured height with ischemic stroke subtypes in UKB and CKB were statistically significant (except for presumed cardioembolic stroke in CKB) and similar to the genetic associations in MEGASTROKE in terms of direction: For presumed cardioembolic stroke, the HRs were 1.17 (95% CI 1.07, 1.28; *p* < 0.001; *n* = 495 cases) in UKB and 1.09 (0.99, 1.28; *p* = 0.09; *n* = 410 cases) in CKB; for other subtypes of ischemic stroke in UKB, the HR was 0.96 (95% CI 0.92, 0.99; *p* = 0.02; *n* = 3,203); and for other nonlacunar stroke and lacunar stroke in CKB, they were 0.93 (0.91, 0.96; *p* < 0.001; *n* = 7,503) and 0.96 (0.94, 0.99; *p* = 0.002; *n* = 6,840), respectively (Fig 2, S8 Table).

The associations of genetically determined and measured height with established cardiovascular risk factors, anthropometric traits, and education are shown in Tables 1 and 2 and S9 Table. Almost all of the associations between genetically determined height and risk factors were directionally concordant and broadly consistent between UKB and CKB, the exceptions being the following: diabetes, where the CIs were wide and overlapped; smoking, which was not associated in either population; and tertiary education, which was positively associated with genetically determined height in UKB but not associated in CKB (Table 1, S9 Table; the generally lower z-statistics in the genetic comparisons in CKB reflect the smaller number of participants studied). Both genetically determined and measured height were strongly associated with lean body mass (in UKB, 0.5 to 0.6 SD higher lean body mass per 1 SD taller genetically determined height, z = 98 [*p* < 0.001] in men, z = 87 [*p* < 0.001] in women) and with lung function (0.3 to 0.4 SD higher FEV1 or FVC, z = 50 to 65 [*p* < 0.001]).

**Table 1:** Associations of genetically determined height with cardiovascular risk factors and anthropometric traits in UKB and CKB.

Baseline characteristic	UKB (*n* = 336,750)			CKB (*n* = 53,346)			Directional consistency^†^
	Effect (95% CI) per 1 SD genetically determined taller height*	Z-statistic	*p*-Value	Effect (95% CI) per 1 SD genetically determined taller height*	Z-statistic	*p*-Value	
**Diagnosed prior disease (OR)**							
Diabetes	0.96 (0.93, 1.00)	−2.0	0.05	1.02 (0.93, 1.12)	0.5	0.65	−+
Atrial fibrillation	1.33 (1.25, 1.42)	8.8	<0.001	NA	NA	NA	NA
Hypertension	0.91 (0.90, 0.93)	−9.6	<0.001	0.97 (0.90, 1.04)	−0.9	0.37	−−
**Blood pressure (mm Hg)**							
Systolic blood pressure	−1.13 (−1.27, −0.98)	−15.1	<0.001	−0.14 (−0.57, 0.29)	−0.6	0.55	−−
Diastolic blood pressure	−0.42 (−0.51, −0.34)	−10.0	<0.001	−0.14 (−0.37, 0.10)	−1.1	0.28	−−
**Blood lipids**							
LDL cholesterol (mmol/L)	−0.042 (−0.049, −0.035)	−11.1	<0.001	−0.055 (−0.103, −0.007)	−2.1	0.03	−−
HDL cholesterol (mmol/L)	−0.007 (−0.010, −0.004)	−4.6	<0.001	−0.006 (−0.028, 0.016)	−0.5	0.61	−−
Triglycerides (mmol/L)	−0.037 (−0.046, −0.029)	−8.5	<0.001	−0.061 (−0.172, 0.050)	−1.0	0.30	−−
Apolipoprotein B (g/L)	−0.014 (−0.016, −0.012)	−13.5	<0.001	−0.019 (−0.034, −0.005)	−2.5	0.01	−−
**Lung function (SD units within sex)**							
FEV1, men	0.312 (0.300, 0.325)	49.8	<0.001	0.220 (0.193, 0.247)	15.2	<0.001	++
FEV1, women	0.295 (0.284, 0.307)	51.5	<0.001	0.221 (0.200, 0.243)	19.0	<0.001	++
FVC, men	0.414 (0.402, 0.427)	64.6	<0.001	0.268 (0.240, 0.296)	17.7	<0.001	++
FVC, women	0.382 (0.371, 0.394)	65.2	<0.001	0.259 (0.236, 0.282)	21.4	<0.001	++
**Anthropometric measures (SD units within sex)**							
BMI, men	−0.070 (−0.083, −0.058)	−11.2	<0.001	−0.054 (−0.086, −0.021)	−3.1	0.002	−−
BMI, women	−0.073 (−0.085, −0.062)	−12.6	<0.001	−0.062 (−0.088, −0.037)	−4.5	<0.001	−−
Waist to hip, men	−0.023 (−0.035, −0.011)	−3.8	<0.001	−0.041 (−0.076, −0.007)	−2.2	0.03	−−
Waist to hip, women	−0.021 (−0.033, −0.010)	−3.7	<0.001	−0.007 (−0.032, 0.018)	−0.5	0.61	−−
Weight, men	0.397 (0.385, 0.409)	64.0	<0.001	0.337 (0.306, 0.367)	20.8	<0.001	++
Weight, women	0.322 (0.310, 0.333)	55.7	<0.001	0.343 (0.319, 0.367)	26.5	<0.001	++
Lean body mass, men	0.589 (0.578, 0.601)	98.0	<0.001	0.488 (0.458, 0.518)	30.1	<0.001	++
Lean body mass, women	0.492 (0.480, 0.503)	86.9	<0.001	0.598 (0.574, 0.621)	47.9	<0.001	++

In UKB, blood lipids measurements were available in 87% to 95% of participants, lung function in 76%, and anthropometric traits in ≥98% (S1 Fig). In CKB, blood lipids measurements were available in 8% of participants and lung function in 82% (S2 Fig).

*Effects are the ORs for prior disease or the difference in the characteristic per 1 SD genetically determined taller height, adjusted for age, age^2^, sex, region (in CKB only), genomic principal components, and genotyping array type. For UKB and CKB, respectively, the SDs of directly measured height were 6.8 cm versus 6.5 cm for men and 6.3 cm versus 6.0 cm for women.

^†^Each pair of signs indicates the direction of the estimated effect for UKB (first sign) and CKB (second sign).

BMI, body mass index; CI, confidence interval; CKB, China Kadoorie Biobank; FEV1, forced expiratory volume in 1 second; FVC, forced vital capacity; HDL, high-density lipoprotein; LDL, low-density lipoprotein; NA, not available; OR, odds ratio; SD, standard deviation; UKB, UK Biobank.

**Table 2:** Associations of measured height with cardiovascular risk factors and anthropometric traits in UKB and CKB.

Baseline characteristic	UKB (*n* = 482,928)			CKB (*n* = 490,067)			Directional consistency^†^
	Effect (95% CI) per 1 SD taller measured height*	Z-statistic	*p*-Value	Effect (95% CI) per 1 SD taller measured height*	Z-statistic	*p*-Value	
**Diagnosed prior disease (OR)**							
Diabetes	0.85 (0.84, 0.86)	−28.5	<0.001	1.06 (1.04, 1.07)	8.4	<0.001	−+
Atrial fibrillation	1.31 (1.28, 1.34)	22.2	<0.001	NA	NA	NA	NA
Hypertension	0.87 (0.87, 0.88)	−41.6	<0.001	1.13 (1.12, 1.14)	24.8	<0.001	−+
**Blood pressure (mm Hg)**							
Systolic blood pressure	−1.16 (−1.21, −1.11)	−46.4	<0.001	0.32 (0.27, 0.38)	11.7	<0.001	−+
Diastolic blood pressure	−0.36 (−0.39, −0.33)	−25.3	<0.001	0.44 (0.41, 0.47)	28.5	<0.001	−+
**Blood lipids**							
LDL cholesterol (mmol/L)	−0.017 (−0.019, −0.014)	−13.0	<0.001	0.008 (−0.001, 0.018)	1.7	0.10	−+
HDL cholesterol (mmol/L)	0.005 (0.004, 0.007)	10.2	<0.001	−0.012 (−0.016, −0.008)	−5.8	<0.001	+−
Triglycerides (mmol/L)	−0.047 (−0.050, −0.044)	−31.7	<0.001	0.049 (0.026, 0.071)	4.3	<0.001	−+
Apolipoprotein B (g/L)	−0.009 (−0.009, −0.008)	−24.6	<0.001	0.003 (0.000, 0.006)	2.3	0.02	−+
**Lung function (SD units within sex)**							
FEV1, men	0.370 (0.366, 0.374)	173.7	<0.001	0.281 (0.278, 0.284)	168.7	<0.001	++
FEV1, women	0.355 (0.352, 0.359)	188.1	<0.001	0.280 (0.277, 0.282)	197.9	<0.001	++
FVC, men	0.452 (0.448, 0.456)	216.4	<0.001	0.311 (0.308, 0.315)	186.6	<0.001	++
FVC, women	0.425 (0.421, 0.429)	227.3	<0.001	0.305 (0.302, 0.307)	215.1	<0.001	++
**Anthropometric measures (SD units within sex)**							
BMI, men	−0.056 (−0.060, −0.051)	−25.8	<0.001	0.040 (0.036, 0.044)	19.1	<0.001	−+
BMI, women	−0.119 (−0.123, −0.115)	−61.9	<0.001	0.002 (−0.002, 0.005)	0.9	0.36	−+
Waist to hip, men	−0.048 (−0.052, −0.044)	−22.6	<0.001	0.043 (0.039, 0.047)	19.9	<0.001	−+
Waist to hip, women	−0.075 (−0.079, −0.071)	−39.3	<0.001	0.003 (−0.001, 0.006)	1.5	0.13	−+
Weight, men	0.410 (0.406, 0.414)	208.9	<0.001	0.436 (0.433, 0.440)	253.2	<0.001	++
Weight, women	0.274 (0.270, 0.277)	146.9	<0.001	0.407 (0.404, 0.410)	263.7	<0.001	++
Lean body mass, men	0.608 (0.605, 0.611)	363.3	<0.001	0.586 (0.583, 0.589)	384.1	<0.001	++
Lean body mass, women	0.470 (0.466, 0.473)	276.8	<0.001	0.650 (0.648, 0.652)	555.4	<0.001	++

In UKB, blood lipids measurements were available in 85% to 93% of participants, lung function in 71%, and anthropometric traits in ≥98% (S1 Fig). In CKB, blood lipids measurements were available in 4% of participants and lung function in 87% (S2 Fig).

*Effects are the ORs for prior disease or the difference in the characteristic per 1 SD taller measured height, adjusted for age (in 5-year groups), sex, year of birth, and region (in CKB only).

^†^Each pair of signs indicates the direction of the estimated effect for UKB (first sign) and CKB (second sign).

BMI, body mass index; CI, confidence interval; CKB, China Kadoorie Biobank; FEV1, forced expiratory volume in 1 second; FVC, forced vital capacity; HDL, high-density lipoprotein; LDL, low-density lipoprotein; NA, not available; OR, odds ratio; SD, standard deviation; UKB, UK Biobank.

Genetically determined taller height was also associated with lower levels of LDL cholesterol, HDL cholesterol, and blood pressure in UKB and nonstatistically significant lower levels in CKB; however, the estimated effect sizes on blood pressure were greater in UKB than in CKB and the CIs of the estimates did not overlap (−1.13 mm Hg [95% CI −1.27, −0.98; *p* < 0.001] versus −0.14 mm Hg [95% CI −0.57, 0.29; *p* = 0.55]). In UKB, the findings for measured and genetically determined height with systolic blood pressure were highly consistent (Tables 1 and 2), but in CKB, the measured height was positively, rather than inversely, associated with systolic blood pressure, suggesting that this association might reflect confounding in CKB. Both genetically determined and measured height were strongly positively associated with atrial fibrillation at baseline (available only in UKB) with ORs per 1 SD taller height of 1.33 (95% CI 1.25, 1.42; *p* < 0.001) and 1.31 (1.28, 1.34; *p* < 0.001), respectively (Tables 1 and 2).

## Discussion

In this large MR study of height and ischemic stroke, there were modest inverse associations of both genetically determined and measured height with overall ischemic stroke in populations from multiple ancestries. However, these masked much stronger directionally opposing associations of height with cardioembolic versus other ischemic stroke subtypes. In MEGASTROKE (multiple ancestries), a 1 SD genetically determined taller height was associated with 13% higher risk (OR 1.13 [95% CI 1.07, 1.19]; *p* < 0.001) of cardioembolic stroke, but with 11% lower (OR 0.89 [0.84, 0.95]; *p* < 0.001) and 13% lower (OR 0.87 [0.83, 0.92]; *p* < 0.001) risks of large-artery stroke and small-vessel stroke, respectively. In UKB and CKB, the different associations of measured height with ischemic stroke subtypes were concordant with those in MEGASTROKE. However, the genetic associations in UKB and CKB, although consistent, had less power to reliably demonstrate differences between the different ischemic stroke subtypes. Nevertheless, the similar findings from observational and MR approaches across 3 different populations provide support for height being causally related to ischemic stroke subtypes.

To the best of our knowledge, this is the first large genetic study to examine the associations of height with ischemic stroke subtypes and furthermore included multiple ancestries. A previous study reported an OR of 0.88 (95% CI 0.82, 0.95) per 1 SD taller genetically determined height with ischemic heart disease [4], which is similar to association with large-artery stroke in the present study and could be a reflection of a shared underlying process affecting height and atherosclerosis. The present study used MR approaches that minimize biases from residual confounding and reverse causality that can bias observational studies. Furthermore, in a range of MR sensitivity analyses, the findings remained consistent irrespective of the methodology used for estimation and found no evidence to support any major influence of horizontal pleiotropy. For example, the associations of genetically determined height with the stroke subtypes remained similar when SNPs most strongly associated (at *p* < 0.001) with length of education, LDL cholesterol, blood pressure and other cardiovascular risk factors were excluded from the genetic instrument.

The modest impact of excluding SNPs most strongly associated with cardiovascular risk factors suggests that any mediating effect of such traits is likely to be low. However, LDL cholesterol has previously been shown to be causally associated with increased risk of ischemic stroke in populations of both European and Chinese ancestries [21], with the strongest association observed with large-artery stroke and little association seen with cardioembolic stroke [27]. Thus, the inverse association of genetically determined height with LDL cholesterol levels in both UKB and CKB could explain some of the inverse associations of height with large-artery stroke and, to a lesser extent, with small-vessel stroke, although the mechanism by which height might cause this is unclear. Genetically determined taller height was also associated with lower mean levels of blood pressure in both studies (about 1 mm Hg lower in UKB, but only 0.1 mm Hg in CKB; Table 1); based on the UKB effect, this would be expected to translate to about 3% proportional lower risk of ischemic stroke and 2% to 5% proportional lower risk of each ischemic stroke subtype [28]. By contrast with the consistency of the genetic associations, the observational associations were not as consistent between UKB and CKB, possibly reflecting differences in residual confounding in the observational analyses (e.g., by socioeconomic factors, as blood pressure and height are positively correlated with income in China [29]) or reverse causality (e.g., due to LDL-lowering medication), illustrating the advantage of MR analyses.

The associations of height with ischemic stroke subtypes may reflect a direct causal effect of body dimensions on stroke subtypes or the effects of some other correlated anthropometric trait (such as lean body mass) on the diseases. Previous MR studies have suggested that greater lung function may act as a possible mediator of the protective effect of height on ischemic heart disease [5]. In both UKB and CKB, taller height was associated with higher lung function and so lung function could account for some of the protective effects of height [5].

This study provides novel support for the causal relevance of height for cardioembolic stroke, the most disabling consequence of atrial fibrillation. Previous studies have supported the causal relevance of height and lean body mass for atrial fibrillation [6,7] and suggested that greater lean body mass is the chief anthropometric risk factor (stronger than height) for atrial fibrillation [7]. Larger left atrial diameter, present in taller people, has also been associated with higher risks of atrial fibrillation and embolism from cardiac sources [30], but whether these associations are mediated by lean body mass or some other physical aspect of body dimensions has not been previously studied. Higher levels of lean body mass have also been positively associated with other physical measures, such as carotid intima-media thickness, left ventricular mass, and cardiac wall thickness, but not with atherosclerosis [31].

The opposing associations of height with cardioembolic and other ischemic stroke subtypes highlight the importance of considering ischemic stroke subtypes as distinct diseases. Studies examining the associations of risk factors with overall ischemic stroke may incorrectly estimate medically relevant associations of some risk factors with individual ischemic stroke subtypes. Many studies (e.g., UKB, with follow-up based on electronic health records) and cardiovascular trials do not currently have detailed and reliable ischemic stroke subtyping, limiting their use for causal inference. Subtyping is also important in clinical practice for prevention of stroke recurrence, where the impact of treatments, such as statins or anticoagulants, may vary in patients at particular risk for different ischemic stroke subtypes [27].

Men and women in CKB were 10 and 8 cm shorter (about 1.5 SD), respectively, than their counterparts in UKB (S1 Table). If the MR associations in Fig 2 are assumed to be causal, this would translate to adults in China having a higher risk of some ischemic stroke subtypes (particularly for large-artery stroke and small-vessel stroke subtypes) and a lower risk of cardioembolic stroke compared with Europeans. In CKB, genetically determined height was associated with a modestly, albeit not statistically, significant lower OR for all ischemic stroke subtypes.

The present study also had several limitations. Genotypes associated with height, education, blood pressure, and several chronic diseases have been shown to be correlated within spouse pairs (i.e., indicative of assortative mating), which can lead to indirect effects of genotypes in offspring, in violation of MR assumptions [32]. Family-based studies have reported that such indirect genetic effects of nontransmitted alleles could explain about 12% of the genetic effect on height [33]. As desirable traits such as higher income, taller height, and healthy traits tend to cluster in mates, assortative mating could explain some of the protective associations of taller height, but is unlikely to explain the adverse associations of height with atrial fibrillation and cardioembolic stroke.

A further limitation is that studies differed in the methodology used to classify ischemic stroke subtypes, and reliable subtyping was not available in all of the populations studied. As cardioembolic stroke has been reported to account for 22% of ischemic stroke cases in a global meta-analysis [34] and over half of cases in a Canadian registry study [35], the relatively low number of presumed cardioembolic stroke cases observed in both UKB and CKB may be an underestimate of the true incidence of cardioembolic strokes.

While height has been estimated to have a SNP-based heritability of about 50% in both Europeans [19] and East Asians [20], it is likely that genetic instruments derived in European populations may not perform as well in other ancestry populations, due to differences in allele frequencies and LD structure, but can still provide valid causal inferences [21,22]. The genetic risk scores for height used in UKB (based on an independent largely European ancestry-based GWAS) explained 19.7% of the variance in height in UKB, but the genetic risk score used in CKB (based on a large GWAS of height in a European population [18] and a smaller GWAS of height in a Japanese population) [20] explained only 15.2% of the variance in height in CKB. The present multiple ancestry analysis in MEGASTROKE may therefore have underestimated the causal effects of height if the (European ancestry derived) genetic risk score used was associated with smaller differences in height in the non-European ancestry populations.

The findings in the present study highlight important differences in the causal pathways between stroke subtypes and the need to distinguish such subtypes not only in clinical practice, but also in cardiovascular trials, electronic health records, and population studies. Although height is not a modifiable risk factor, recognition that taller individuals have increased risk of cardioembolic stroke may guide clinicians to screen for atrial fibrillation or other risk factors for cardioembolic stroke when managing an individual’s overall risk [3]. Further research is needed to understand the shared biological and physical pathways underlying the associations of height with stroke subtypes. The strong association of genetically determined height with physical measurements such as lean body mass and lung function and with atrial fibrillation suggest that these may be mediators of some of the associations with height. Further study, such as multivariable MR with robust instruments (probably sex specific, because of the substantial differences in anthropometric measures by sex), could yield further insight into the direct and indirect effects of height through other factors on the risks of ischemic stroke subtypes.

In conclusion, the present genetic studies provide novel and reliable findings that support a causal association of taller adult height with higher risks of atrial fibrillation and cardioembolic stroke and lower risks of other ischemic stroke subtypes. These findings raise the possibility of investigating whether including height as a risk factor in risk prediction tools would improve screening and primary prevention of cardioembolic stroke and of whether understanding the shared biological and physical pathways involved in height may offer novel targets for treatment to prevent cardioembolic stroke.

## Supporting information

S1 Text Members of the CKB Collaborative Group. CKB, China Kadoorie Biobank.
(DOCX)

S2 Text Members of the MEGASTROKE consortium. (DOCX)

S1 Checklist STROBE-MR checklist of recommended items to address in reports of MR studies. This checklist is copyrighted by the Equator Network under the Creative Commons Attribution 3.0 Unported (CC BY 3.0) license. MR, mendelian randomization; NA, not applicable; STROBE-MR, Strengthening the Reporting of Observational Studies in Epidemiology using Mendelian Randomization.
(DOCX)

S1 Methods Revisions after study initiation. (DOCX)

S2 Methods Additional methods for UKB. UKB, UK Biobank.
(DOCX)

S3 Methods Additional methods for CKB. CKB, China Kadoorie Biobank.
(DOCX)

S4 Methods Further details of instruments for genetically determined height. (DOCX)

S5 Methods Genetic analyses in MEGASTROKE. (DOCX)

S6 Methods Sensitivity analyses in MEGASTROKE. (DOCX)

S7 Methods Further details of observational analyses in UKB and CKB. CKB, China Kadoorie Biobank; UKB, UK Biobank.
(DOCX)

S1 Table Baseline characteristics of included UKB and CKB participants without prior major cardiovascular disease. Data are *n* (%) or mean (SD) unless otherwise stated. In UKB, blood lipids measurements were available in 85% to 93% of participants, lung function in 71%, and anthropometric traits ≥98% (S1 Fig). In CKB, blood lipids measurements were available in 4% of participants and lung function in 87% (S2 Fig). BMI, body mass index; CKB, China Kadoorie Biobank; FEV1, forced expiratory volume in 1 second; FVC, forced vital capacity; HDL, high-density lipoprotein; IQR, interquartile range; LDL, low-density lipoprotein; NA, not available; UKB, UK Biobank.
(DOCX)

S2 Table Ancestry composition of stroke cases in MEGASTROKE. Ancestry was predominantly self-reported in the 29 genome-wide studies comprising the MEGASTROKE consortium data. *Other ancestry included Latin American and mixed Asian ancestry.
(DOCX)

S3 Table Percentage of height variance explained (R^2^) by genetic instruments for height in UKB and CKB. *Each individual genetic instrument for height, based on GIANT or Biobank Japan SNPs, was linkage disequilibrium pruned (r^2^ < 0.05). ^†^The number of linkage disequilibrium pruned SNPs available in UKB or CKB. ^‡^Beta estimate of measured height regressed on the genetic risk score for height, adjusted for age, age^2^, sex, region (in CKB only), genomic principal components, and genotyping array type. Biobank Japan, Biobank Japan genome-wide association study (2019) [20]; CKB, China Kadoorie Biobank; GIANT (2014), Genetic Investigation of Anthropometric Traits (2014) [19]; GIANT (2018), Genetic Investigation of Anthropometric Traits (2018) [18]; R^2^, the proportion of the residual variance of height explained by the genetic risk score for height (the coefficient of determination); SNP, single nucleotide polymorphism; UKB, UK Biobank.
(DOCX)

S4 Table Associations of the genetic risk score for height with ischemic stroke and its subtypes in UKB and CKB. CKB, China Kadoorie Biobank; UKB, UK Biobank.
(DOCX)

S5 Table Weighted median, MR–Egger, and MR–PRESSO sensitivity analyses of the associations of genetically determined height with ischemic stroke and its subtypes in MEGASTROKE. *OR per 1 SD genetically determined taller height. The numbers of events reported for MEGASTROKE were the maximum number of cases available in the genetic summary data. GIANT (2018), Genetic Investigation of Anthropometric Traits (2018) [18]; IVW, inverse variance weighted; MR, mendelian randomization; MR–PRESSO, Mendelian Randomization Pleiotropy RESidual Sum and Outlier; OR, odds ratio; SD, standard deviation.
(DOCX)

S6 Table Additional sensitivity analyses of the associations of genetically determined height with ischemic stroke and its subtypes in MEGASTROKE using (A) a restricted genetic instrument excluding SNPs associated with age at completion of full-time education, diabetes, atrial fibrillation, hypertension, systolic blood pressure, diastolic blood pressure, LDL cholesterol, HDL cholesterol, triglycerides, or apolipoprotein B and (B) a genetic instrument for height with a stricter level of LD pruning (r^2^ < 0.001). *The number of SNPs available for each stroke subtype varied from 2,084 to 2,277 in the main genetic instrument for height, from 1,377 to 1,514 in the restricted genetic instrument in (A) and from 1,114 to 1,180 in the genetic instrument in (B). See S4 Methods (further details of instruments for genetically determined height). ^†^In the pan-ancestry genetic analysis of the UKB (Pan-UKBB) based on 294,072 to 421,391 participants [26]. Percentages of SNPs associated (at *p* < 0.001) with each risk factor were age at completion of full-time education (2.8%), diabetes (2.2%), atrial fibrillation (1.4%), hypertension (7.1%), systolic blood pressure (10.3%), diastolic blood pressure (8.9%), LDL cholesterol (5.7%), HDL cholesterol (10.7%), triglycerides (10.5%), and apolipoprotein B (6.5%). HDL, high-density lipoprotein; LD, linkage disequilibrium; LDL, low-density lipoprotein; OR, odds ratio; SNP, single nucleotide polymorphism; UKB, UK Biobank.
(DOCX)

S7 Table Associations of genetically determined height with ischemic stroke and its subtypes in CKB shown for different genetic instruments. Each individual genetic instrument for height, based on GIANT or Biobank Japan SNPs, was linkage disequilibrium pruned (r^2^ < 0.05). The category “All ischemic stroke” includes additional unsubtyped ischemic strokes. Genetic associations in CKB were adjusted for age, age^2^, sex, region, genomic principal components, and genotyping array type. Biobank Japan, Biobank Japan genome-wide association study (2019) [20]; CKB, China Kadoorie Biobank; GIANT (2018), Genetic Investigation of Anthropometric Traits (2018) [18]; OR, odds ratio; R^2^, the proportion of the residual variance of height explained by the genetic risk score for height (the coefficient of determination); SNP, single nucleotide polymorphism.
(DOCX)

S8 Table Associations of measured height with ischemic stroke and its subtypes in UKB and CKB. For UKB and CKB, respectively, the SDs of directly measured height were 6.8 cm versus 6.5 cm for men and 6.3 cm versus 6.0 cm for women. *Associations were stratified by age at risk (in 5-year groups), sex, and region (in CKB only) and adjusted for year of birth. ^†^Additional potential confounders included year of birth, smoking status, number of cigarettes smoked, systolic blood pressure, diastolic blood pressure, diagnosed hypertension, diagnosed diabetes, self-rated walking pace (UKB only), and level of education (S6 Methods). CKB, China Kadoorie Biobank; HR, hazard ratio; SD, standard deviation; UKB, UK Biobank.
(DOCX)

S9 Table Associations of genetically determined height with other cardiovascular risk factors—Smoking status and education. *Effects are the ORs per 1 SD genetically determined taller height, adjusted for age, age^2^, sex, region (in CKB only), genomic principal components, and genotyping array type. For UKB and CKB, respectively, the SDs of directly measured height were 6.8 cm versus 6.5 cm for men and 6.3 cm versus 6.0 cm for women. ^†^Each pair of signs indicates the direction of the estimated effect for UKB (first sign) and CKB (second sign). CKB, China Kadoorie Biobank; OR, odds ratio; SD, standard deviation; UKB, UK Biobank.
(DOCX)

S1 Fig Data flow diagram for UKB. UKB, UK Biobank.
(TIF)

S2 Fig Data flow diagram for CKB. CKB, China Kadoorie Biobank.
(TIF)

S3 Fig MR framework for MEGASTROKE, UKB, and CKB. Biobank Japan, Biobank Japan genome-wide association study (2019) [20]; CKB, China Kadoorie Biobank; GC, genetic consortia (which differs between studies); GIANT (2014), Genetic Investigation of Anthropometric Traits (2014) [19]; GIANT (2018), Genetic Investigation of Anthropometric Traits (2018) [18]; GWAS, genome-wide association study; HR, hazard ratio; MR, mendelian randomization; OR, odds ratio; SNP, single nucleotide polymorphism; UKB, UK Biobank.
(TIF)

S4 Fig Effects of height-associated SNPs on ischemic stroke and its subtypes in MEGASTROKE (multiple ancestry) in the relation to their effects on height in GIANT (2018). For MEGASTROKE (multiple ancestry), 2,265 height-associated SNPs were available for ischemic stroke cases, 2,270 for cardioembolic and large-artery stroke cases, and 2,084 for small-vessel stroke cases. GIANT (2018), Genetic Investigation of Anthropometric Traits (2018) [18]; SD, standard deviation; SNP, single nucleotide polymorphism;.
(TIF)

S1 Data Table SNPs used to construct the genetic instrument for height in MEGASTROKE. *GIANT (2018), Genetic Investigation of Anthropometric Traits (2018) [18]. SNP, single nucleotide polymorphism.
(XLSX)

S2 Data Table SNPs used to construct the genetic instrument for height in UKB. *GIANT (2014), Genetic Investigation of Anthropometric Traits (2014) [19]. SNP, single nucleotide polymorphism; UKB, UK Biobank.
(XLSX)

S3 Data Table SNPs used to construct the genetic instrument for height in CKB. *GIANT (2018), Genetic Investigation of Anthropometric Traits (2018) [18]. ^†^Biobank Japan genome-wide association study (2019) [20]. CKB, China Kadoorie Biobank; SNP, single nucleotide polymorphism.
(XLSX)

S4 Data Table Associations of SNPs used to construct the genetic instrument for height in MEGASTROKE with ischemic stroke and its subtypes. SNP, single nucleotide polymorphism.
(XLSX)

## Abbreviations

BMI: body mass index
CI: confidence interval
CKB: China Kadoorie Biobank
FEV1: forced expiratory volume in 1 second
FVC: forced vital capacity
GIANT: Genetic Investigation of Anthropometric Traits
GWAS: genome-wide association study
HDL: high-density lipoprotein
HR: hazard ratio
LD: linkage disequilibrium
LDL: low-density lipoprotein
MR: mendelian randomization
MR–PRESSO: Mendelian Randomization Pleiotropy RESidual Sum and Outlier
OR: odds ratio
SD: standard deviation
SNP: single nucleotide polymorphism
STROBE-MR: Strengthening the Reporting of Observational Studies in Epidemiology using Mendelian Randomization
TOAST: Trial of ORG 10 172 in Acute Stroke Treatment
UKB: UK Biobank
