# Supplementary material for: Genetic associations of adult height with risk of cardioembolic and other subtypes of ischemic stroke: A mendelian randomization study in multiple ancestries
Source: PLoS Med. 2022 Apr 22;19(4):e1003967. doi: 10.1371/journal.pmed.1003967 (PMC9032370; doi:10.1371/journal.pmed.1003967)
Supplement: S1 Text — CKB, China Kadoorie Biobank. (DOCX) [file pmed.1003967.s001.docx]

## S1 Text. Members of the China Kadoorie Biobank Collaborative Group.

***International steering committee:*** Junshi Chen, Zhengming Chen (PI), Robert Clarke, Rory Collins, Yu Guo, Liming Li (PI), Jun Lv, Richard Peto, and Robin Walters.

***International co-ordinating centre, Oxford:*** Daniel Avery, Derrick Bennett, Ruth Boxall, Fiona Bragg, Yumei Chang, Yiping Chen, Zhengming Chen, Robert Clarke, Huaidong Du, Simon Gilbert, Alex Hacker, Michael Holmes, Christiana Kartsonaki, Rene Kerosi, Garry Lancaster, Kuang Lin, John McDonnell, Iona Millwood, Qunhua Nie, Jayakrishnan Radhakrishnan, Paul Ryder, Sam Sansome, Dan Schmidt, Rajani Sohoni, Iain Turnbull, Robin Walters, Jenny Wang, Lin Wang, Neil Wright, Ling Yang, and Xiaoming Yang.

***National co-ordinating centre, Beijing:*** Zheng Bian, Yu Guo, Xiao Han, Can Hou, Biao Jing, Chao Liu, Jun Lv, Pei Pei, Yunlong Tan, and Canqing Yu.

***Regional co-ordinating centres:*** **Qingdao** Qingdao CDC: Zengchang Pang, Ruqin Gao, Shanpeng Li, Shaojie Wang, Yongmei Liu, Ranran Du, Yajing Zang, Liang Cheng, Xiaocao Tian, Hua Zhang, Yaoming Zhai, Feng Ning, Xiaohui Sun, Feifei Li. Licang CDC: Silu Lv, Junzheng Wang, Wei Hou. **Heilongjiang** Provincial CDC: Mingyuan Zeng, Ge Jiang, Xue Zhou. Nangang CDC: Liqiu Yang, Hui He, Bo Yu, Yanjie Li, Qinai Xu, Quan Kang, Ziyan Guo. **Hainan** Provincial CDC: Dan Wang, Ximin Hu, Hongmei Wang, Jinyan Chen, Yan Fu, Zhenwang Fu, Xiaohuan Wang. Meilan CDC: Min Weng, Zhendong Guo, Shukuan Wu, Yilei Li, Huimei Li, Zhifang Fu. **Jiangsu** Provincial CDC: Ming Wu, Yonglin Zhou, Jinyi Zhou, Ran Tao, Jie Yang, Jian Su. Suzhou CDC: Fang Liu, Jun Zhang, Yihe Hu, Yan Lu, Liangcai Ma, Aiyu Tang, Shuo Zhang, Jianrong Jin, Jingchao Liu. **Guangxi** Provincial CDC: Zhenzhu Tang, Naying Chen, Ying Huang. Liuzhou CDC: Mingqiang Li, Jinhuai Meng, Rong Pan, Qilian Jiang, Jian Lan, Yun Liu, Liuping Wei, Liyuan Zhou, Ningyu Chen, Ping Wang, Fanwen Meng, Yulu Qin, Sisi Wang. **Sichuan** Provincial CDC: Xianping Wu, Ningmei Zhang, Xiaofang Chen, Weiwei Zhou. Pengzhou CDC: Guojin Luo, Jianguo Li, Xiaofang Chen, Xunfu Zhong, Jiaqiu Liu, Qiang Sun. **Gansu** Provincial CDC: Pengfei Ge, Xiaolan Ren, Caixia Dong. Maiji CDC: Hui Zhang, Enke Mao, Xiaoping Wang, Tao Wang, Xi Zhang. **Henan** Provincial CDC: Ding Zhang, Gang Zhou, Shixian Feng, Liang Chang, Lei Fan. Huixian CDC: Yulian Gao, Tianyou He, Huarong Sun, Pan He, Chen Hu, Xukui Zhang, Huifang Wu. **Zhejiang** Provincial CDC: Min Yu, Ruying Hu, Hao Wang. Tongxiang CDC: Yijian Qian, Chunmei Wang, Kaixu Xie, Lingli Chen, Yidan Zhang, Dongxia Pan, Qijun Gu. **Hunan** Provincial CDC: Yuelong Huang, Biyun Chen, Li Yin, Huilin Liu, Zhongxi Fu, Qiaohua Xu. Liuyang CDC: Xin Xu, Hao Zhang, Huajun Long, Xianzhi Li, Libo Zhang, and Zhe Qiu.
