# Supplementary material for: Genetic associations of adult height with risk of cardioembolic and other subtypes of ischemic stroke: A mendelian randomization study in multiple ancestries
Source: PLoS Med. 2022 Apr 22;19(4):e1003967. doi: 10.1371/journal.pmed.1003967 (PMC9032370; doi:10.1371/journal.pmed.1003967)
Supplement: S1 Methods — (DOCX) [file pmed.1003967.s004.docx]

## S1 Methods. Revisions after study initiation.

The present study was initiated with the publication of the MEGASTROKE data in 2018 [1]. Initially, the genetic instruments for height were constructed only from genome-wide association studies (GWAS) summary statistics for height published by the Genetic Investigation of Anthropometric Traits (GIANT) Consortium (comprising individuals mainly of European ancestry) [2,3]. However, genetic instruments derived in European populations do not perform as well in other ancestry populations [4,5]. Therefore, when the GWAS summary statistics for height from the Biobank Japan study (comprising individuals of East Asian ancestry) [6] were published in 2019, these summary statistics were included in the construction of the genetic instrument for height in the China Kadoorie Biobank (CKB). As described in the Methods section (*Instruments for genetically-determined height*) and shown in S3 Table, in addition to the method adopted, other weightings of the GIANT-based and Biobank-Japan-based instruments were considered. Based on comments from co-authors, colleagues and peer review: (i) the present study’s analyses were revised to replace palindromic single nucleotide polymorphisms (SNPs) in the genetic instrument for MEGASTROKE with proxies (Methods section *Instruments for genetically-determined height*; palindromic SNPs in UK Biobank (UKB) and CKB were validated by the study teams during their genetic quality control and so no further action was taken on them); (ii) the linkage disequilibrium (LD) pruning level for the height-associated SNPs was also reduced (from r^2^<0.2 to r^2^<0.05); (iii) additional sensitivity analyses were performed in MEGASTROKE, namely weighted median analyses, further analyses using a stricter level of LD pruning (r^2^<0.001) for height-associated SNPs, exclusion of SNPs associated with various other cardiovascular risk factors at a nominal p-value of 0.001 in the pan-ancestry UK Biobank [7] genome-wide analyses and using UKB rather than GIANT as the source of the SNP weightings in the GIANT-based genetic instrument.

## Supplementary references

1. Malik R, Chauhan G, Traylor M, Sargurupremraj M, Okada Y, Mishra A, et al. Multiancestry genome-wide association study of 520,000 subjects identifies 32 loci associated with stroke and stroke subtypes. Nat Genet. 2018;50: 524–537. doi:10.1038/s41588-018-0058-3

2. Yengo L, Sidorenko J, Kemper KE, Zheng Z, Wood AR, Weedon MN, et al. Meta-analysis of genome-wide association studies for height and body mass index in ∼700000 individuals of European ancestry. Hum Mol Genet. 2018;27: 3641–3649. doi:10.1093/hmg/ddy271

3. Wood AR, Esko T, Yang J, Vedantam S, Pers TH, Gustafsson S, et al. Defining the role of common variation in the genomic and biological architecture of adult human height. Nat Genet. 2014;46: 1173–1186.

4. Sun L, Clarke R, Bennett D, Guo Y, Walters RG, Hill M, et al. Causal associations of blood lipids with risk of ischemic stroke and intracerebral hemorrhage in Chinese adults. Nat Med. 2019;25: 569–574. doi:10.1038/s41591-019-0366-x

5. Martin AR, Kanai M, Kamatani Y, Okada Y, Neale BM, Daly MJ. Clinical use of current polygenic risk scores may exacerbate health disparities. Nat Genet. 2019;51: 584–591. doi:10.1038/s41588-019-0379-x

6. Akiyama M, Ishigaki K, Sakaue S, Momozawa Y, Horikoshi M, Hirata M, et al. Characterizing rare and low-frequency height-associated variants in the Japanese population. Nat Commun. 2019;10: 4393. doi:10.1038/s41467-019-12276-5

7. Pan-ancestry UK Biobank (Pan-UKBB). [cited 16 Dec 2021]. Available: https://pan.ukbb.broadinstitute.org/
