# Supplementary material for: Genetic associations of adult height with risk of cardioembolic and other subtypes of ischemic stroke: A mendelian randomization study in multiple ancestries
Source: PLoS Med. 2022 Apr 22;19(4):e1003967. doi: 10.1371/journal.pmed.1003967 (PMC9032370; doi:10.1371/journal.pmed.1003967)
Supplement: S2 Methods — UKB, UK Biobank. (DOCX) [file pmed.1003967.s005.docx]

## S2 Methods. Additional methods for UK Biobank.

## Baseline measurements in UK Biobank

Standard operating procedures were used to collect information using self-completed questionnaires on demographic, socio-economic, medical history, dietary habits, and lifestyle factors. Height was measured using a Seca 202 device in participants after they were asked to remove their shoes [1]. Measurements were recorded using analogue rulers and entered into the computer system, which flagged implausible values. Weight and percentage body fat were measured using a bio-impedance device (Tanita BC418MA Body Composition Analyser). Blood and urine samples were collected for genetic and plasma biochemistry analyses. “Best measure” lung function measures (forced vital capacity and forced expiratory volume in 1 second) were defined by UKB as the highest measure of a series of lung function measurements which were assessed as acceptable by the investigator. “Best measure” lung function measures were used in the analyses (S1 Fig).

## Adjudication of ischaemic stroke cases in UK Biobank

Participants were linked to death registries and hospital admission records and followed up until 31 March 2017 in England, 31 October 2016 in Scotland, and 29 February 2016 in Wales. The UKB outcome adjudication group have classified selected disease outcomes (including ischaemic stroke) as algorithmically-defined outcomes where, based on pre-defined rules (algorithms), the available evidence supports a high positive predictive value for that outcome [2]. The algorithms used self-reported data collected at baseline (self-reported medical conditions, operations and medications), linked hospital admissions data (diagnoses and procedures), and death registry data. The present analyses used algorithmically-defined ischaemic stroke cases (UKB data-field 42 008).

## Genotyping and imputation in UK Biobank

In UKB, 49 950 participants were genotyped using Affymetrix UK BiLEVE Axiom array (~807 000 variants) and 438 427 were genotyped using an Affymetrix UK Biobank Axiom array (~826 000 variants) in 106 batches [3]. Genotyped data were imputed into both the Haplotype Reference Consortium reference panel and a merged version of the UK10K and the 1000 Genomes phase 3 reference panels, yielding 96 million SNPs for analyses.

## Genetic quality control measures in UK Biobank

Participant samples failing quality control performed by the UKB study, which included duplicates, poor quality genetic information (missing rates >2% [i.e. genotyping call rate ≤98%] or unusually high heterozygosity after adjustment for population structure), mismatch between genetic and reported sex, laboratory errors, or withdrawn consent, were excluded. Poor quality SNPs, identified at a batch level using statistical tests for batch effects, plate effects, departures from Hardy-Weinberg equilibrium, sex effects, array effects, and discordance across control replicates were excluded [3].

## Supplementary references

1. UK Biobank: Protocol for a large-scale prospective epidemiological resource. [cited 1 Aug 2020]. Available: https://www.ukbiobank.ac.uk/wp-content/uploads/2011/11/UK-Biobank-Protocol.pdf

2. UK Biobank: Category 42: Algorithmically-defined outcomes. [cited 1 Aug 2020]. Available: http://biobank.ndph.ox.ac.uk/showcase/label.cgi?id=42

3. Bycroft C, Freeman C, Petkova D, Band G, Elliott LT, Sharp K, et al. The UK Biobank resource with deep phenotyping and genomic data. Nature. 2018;562: 203–209. doi:10.1038/s41586-018-0579-z
