# Supplementary material for: Genetic associations of adult height with risk of cardioembolic and other subtypes of ischemic stroke: A mendelian randomization study in multiple ancestries
Source: PLoS Med. 2022 Apr 22;19(4):e1003967. doi: 10.1371/journal.pmed.1003967 (PMC9032370; doi:10.1371/journal.pmed.1003967)
Supplement: S3 Methods — CKB, China Kadoorie Biobank. (DOCX) [file pmed.1003967.s006.docx]

## S3 Methods. Additional methods for China Kadoorie Biobank.

## Baseline measurements in China Kadoorie Biobank

Standard operating procedures were used to collect information on demographic, socio-economic, medical, dietary, and lifestyle information using an interview-administered questionnaire [1]. Height was measured without shoes using a stadiometer. For participants who were in a wheelchair, unable to stand upright, had neck surgery, or had severe curvature of the spine, arm span was used as a proxy for height. Weight and percentage body fat were measured using a bio-impedance device (Tanita BC-418MA Body Composition Analyser). Blood samples were collected for genetic and plasma biochemistry analyses. Baseline diabetes included diagnosed or screen-detected diabetes. Blood lipids were available in a subset of participants (S2 Fig). Since lung function measurements from Qingdao and Haikou provinces were unreliable, they were excluded from the analyses (S2 Fig).

## Adjudication and subtyping of ischaemic stroke cases in China Kadoorie Biobank

Incident cases of stroke were identified by electronic linkage, via a unique identification number, to established registries of major diseases (i.e., stroke, heart disease, cancer and diabetes) and health insurance claims databases for any hospital admissions, and death registries. The present analyses included ischaemic stroke cases recorded during follow-up from enrolment until 31 December 2016 (CKB data release 15). To confirm the accuracy of stroke diagnoses and to enable classification of ischaemic strokes into subtypes, medical records of all reported first episodes of non-fatal stroke cases were sought for disease verification by health care workers and all subsequently verified cases were adjudicated by a panel of specialist physicians in China. All first episodes of reported hospitalised stroke were collated in a central data repository in the co-ordinating centre at Oxford, UK, prior to loading onto a portable computer tablet (“Portable Verification Device”) in relevant batches for each participating hospital. Local healthcare workers visited the hospitals where stroke cases had been reported to retrieve relevant medical records. On retrieval and verification of reported stroke events, key sections of the medical records were photographed using the Portable Verification Device. The information collected included photographs of the admission notes, discharge summary sheets, and reports of key diagnostic tests (including brain CT/MRI results) recorded during their hospital admission. Brain imaging reports were available for over 92% of retrieved stroke cases. For each reported case, the dates of admission and discharge were checked and relevant clinical data verified and recorded electronically using a standardised data entry form. The data collected included primary and secondary diagnoses of stroke at discharge (including stroke pathological types if available), vital status, other events occurring during hospitalisation, diagnostic tests and their results, and all prescribed medication recorded in the notes. The details of the relevant medical records were uploaded onto a secure internet website for subsequent adjudication.

Trained senior neurologists and stroke physicians in China used a secure internet-based Case Adjudication System for clinical Events (i-CASE) system to adjudicate all verified diagnoses using the World Health Organization criteria for stroke (defined as “rapidly developing clinical signs of focal or global disturbance of cerebral function, lasting more than 24 hours or leading to death due a vascular cause”) [2]. Adjudicators reviewed the findings of brain imaging reports (including the presence of haemorrhage or ischaemia, and laterality and location of cerebral lesions) and other relevant clinical information in patients’ medical records to classify strokes into pathological types: ischaemic stroke, lacunar stroke (LACI), intracerebral haemorrhage or subarachnoid haemorrhage. Presumed cardioembolic strokes with major sources of cardioembolism and normal cerebral vascular imaging were identified among confirmed ischaemic stroke cases. Classification was based on criteria defined in the Causative Classification System (CCS) [3], which attempts to determine the most likely ischaemic stroke aetiology in the presence of multiple potential causes. CCS [3] is an automated, web-based version of an enhanced TOAST [4] classification system (Stop Stroke Study[SSS]-TOAST [5]). Lacunar infarcts (ischaemic lesions <15 mm in diameter) were distinguished from non-lacunar infarcts (ischaemic lesions ≥15 mm in diameter) by adjudicating clinicians on inspection of neuroimaging reports [6]. All verified stroke cases that remained unconfirmed after adjudication were referred for final review by both Chinese- and English- speaking clinicians in Oxford.

## Genotyping and imputation in China Kadoorie Biobank

Baseline blood samples were collected from almost all CKB participants and DNA was extracted and stored in boxes of 96 samples typically collected from one, or sometimes two, clinics. The “population representative sample” genotyped comprised randomly selected boxes of DNA samples, or boxes selected as containing samples from the clinics used for the second resurvey, which had been randomly selected as being representative of the overall CKB cohort [7]. In total, 75 791 participants derived from a population representative sample and an additional 6284 ischaemic stroke cases were genotyped in batches of ~50 plates using one of two versions of a custom-designed Affymetrix biobank array for CKB (the first array had ~700 000 variants and the second array had ~803 000). Using ~532 000 variants passing quality control for both array versions, genotyped data were imputed into the 1000 Genomes phase 3 reference panel, SNPs that were not mono-allelic in EAS, yielding 19 million SNPs with info >0.4. Individual genotypes were extracted as dosages of the minor allele according to the imputed probability of each genotype.

## Genetic quality control measures in China Kadoorie Biobank

Participant samples failing quality control performed by the CKB study, which included duplicates, poor quality genetic information (i.e. genotyping call rates ≤95% or unusually high/low heterozygosity), mismatch between genetic and reported sex, or population outliers (in each ancestry group), were excluded. Poor quality SNPs, identified at a batch-level using statistical tests for batch effects, plate effects, or departures from Hardy-Weinberg equilibrium, were excluded. Where appropriate, these tests were conducted in a single ancestry subset or relatedness-pruned subset of participants (i.e. with no pairs of second cousins or closer). The CKB genetics data also excluded SNPs if they were poorly clustered, had no call in ≥10%, had an overall genotyping call rate ≤98% (in batches that passed initial quality control), or had a minor allele frequency deviating significantly from the reference datasets (minor allele frequency difference of >0.2).

## Supplementary references

1. Chen Z, Chen J, Collins R, Guo Y, Peto R, Wu F, et al. China Kadoorie Biobank of 0.5 million people: survey methods, baseline characteristics and long-term follow-up. Int J Epidemiol. 2011;40: 1652–66. doi:10.1093/ije/dyr120

2. The World Health Organization MONICA Project (monitoring trends and determinants in cardiovascular disease): a major international collaboration. WHO MONICA Project Principal Investigators. J Clin Epidemiol. 1988;41: 105–114. doi:10.1016/0895-4356(88)90084-4

3. Ay H, Benner T, Murat AE, Furie KL, Singhal AB, Jensen MB, et al. A Computerized Algorithm for Etiologic Classification of Ischemic Stroke. Stroke. 2007;38: 2979–2984. doi:10.1161/STROKEAHA.107.490896

4. Adams HP, Bendixen BH, Kappelle LJ, Biller J, Love BB, Gordon DL, et al. Classification of subtype of acute ischemic stroke. Definitions for use in a multicenter clinical trial. TOAST. Trial of Org 10172 in Acute Stroke Treatment. Stroke. 1993;24: 35–41. doi:10.1161/01.STR.24.1.35

5. Ay H, Furie KL, Singhal A, Smith WS, Sorensen AG, Koroshetz WJ. An evidence-based causative classification system for acute ischemic stroke. Ann Neurol. 2005;58: 688–697. doi:10.1002/ana.20617

6. Norrving B. Long-term prognosis after lacunar infarction. Lancet Neurol. 2003;2: 238–245.

7. Millwood IY, Walters RG. Collection, Processing, and Management of Biological Samples in Biobank Studies. In: Chen Z, editor. Population Biobank Studies: A Practical Guide. Singapore: Springer; 2020. pp. 77–97. doi:10.1007/978-981-15-7666-9_4
