# Supplementary material for: Genetic associations of adult height with risk of cardioembolic and other subtypes of ischemic stroke: A mendelian randomization study in multiple ancestries
Source: PLoS Med. 2022 Apr 22;19(4):e1003967. doi: 10.1371/journal.pmed.1003967 (PMC9032370; doi:10.1371/journal.pmed.1003967)
Supplement: S4 Methods — (DOCX) [file pmed.1003967.s007.docx]

## S4 Methods. Further details of instruments for genetically-determined height.

The SNPs selected from the GWASs had been identified using either conditional and joint analysis (the GIANT Consortium studies [1,2]) or single-variant analysis (Biobank Japan [3]). A total of 692 height-associated SNPs were genome-wide significant (p<5x10^-8^) in the GIANT (2014) [2] GWAS report, 3290 SNPs in GIANT (2018) [1] (at a revised significance level of p<1x10^-8^), and 547 SNPs from Biobank Japan [3] (p<5x10^-8^). After LD pruning (r^2^<0.05), a total of 641 SNPs were retained from GIANT (2014) [2], 2424 SNPs from GIANT (2018) [1], and 547 SNPs from Biobank Japan [3]. For UKB, all of the 641 pruned SNPs from GIANT (2014) [2] were available for the present analyses. For CKB, 2337 of the pruned SNPs from GIANT (2018) [1] (European ancestry) and 517 of the pruned SNPs from Biobank Japan [3] (East Asian ancestry) were available for the present analyses. For MEGASTROKE, the number of SNPs from GIANT (2018) [1] that were available for the present analyses, after pruning and replacement/removal of palindromic SNPs, differed by ancestry and by the type of stroke outcome. For multiple ancestry, 2265 were available for ischaemic stroke cases, 2270 for cardioembolic and large-artery stroke cases, and 2084 for small-vessel stroke cases. For European ancestry, 2276 were available for ischaemic stroke cases, 2277 for cardioembolic stroke cases, and 2276 for large-artery and small-vessel stroke cases. The allele coding in the height GWAS SNP datasets and the outcome datasets were aligned so that the height effect alleles were consistently defined.

In UKB and CKB, the proportion of the residual variance of height explained by each genetic risk score for height was estimated by the coefficient of determination (R^2^) from a linear regression of height on each of the genetic risk scores (adjusted for age, age^2^, sex, region in CKB, genomic principal components, and genotyping array type).

## Supplementary references

1. Yengo L, Sidorenko J, Kemper KE, Zheng Z, Wood AR, Weedon MN, et al. Meta-analysis of genome-wide association studies for height and body mass index in ∼700000 individuals of European ancestry. Hum Mol Genet. 2018;27: 3641–3649. doi:10.1093/hmg/ddy271

2. Wood AR, Esko T, Yang J, Vedantam S, Pers TH, Gustafsson S, et al. Defining the role of common variation in the genomic and biological architecture of adult human height. Nat Genet. 2014;46: 1173–1186.

3. Akiyama M, Ishigaki K, Sakaue S, Momozawa Y, Horikoshi M, Hirata M, et al. Characterizing rare and low-frequency height-associated variants in the Japanese population. Nat Commun. 2019;10: 4393. doi:10.1038/s41467-019-12276-5
