# Supplementary material for: Genetic associations of adult height with risk of cardioembolic and other subtypes of ischemic stroke: A mendelian randomization study in multiple ancestries
Source: PLoS Med. 2022 Apr 22;19(4):e1003967. doi: 10.1371/journal.pmed.1003967 (PMC9032370; doi:10.1371/journal.pmed.1003967)
Supplement: S5 Methods — (DOCX) [file pmed.1003967.s008.docx]

## S5 Methods. Genetic analyses in MEGASTROKE.

Ancestry was predominantly self-reported in the 29 genome-wide studies comprising the MEGASTROKE consortium data [1]. MEGASTROKE used transethnic meta-analysis of GWAS (MANTRA methodology [2]) to obtain GWAS summary results from participants of multiple ancestries, and fixed-effects meta-analysis across studies involving participants of European ancestry [1]. European was the largest ancestry component in MEGASTROKE (S2 Table) and summary results for separate non-European ancestries were not made available by the consortium. In the present analyses, a two-sample Mendelian randomisation (MR) approach was applied to MEGASTROKE summary data (as individual participant data were not available). The causal effects were estimated by inverse-variance weighted random-effects meta-analyses [3] using: (i) the published single-variant effect sizes on height (in SD units) for each genome-wide significant SNP from the GIANT (2018) [4] GWAS of height (after LD pruning), and (ii) the associations and standard errors (log odds ratios) of each of these height-associated SNPs with ischaemic stroke or ischaemic stroke subtypes from the MEGASTROKE GWAS [1]. The causal effects were presented as ORs (and 95% CIs) for ischaemic stroke subtypes per one standard deviation (1-SD) genetically-determined taller height. This two-sample MR was implemented in R (version 3.3.3) using the MendelianRandomization package. The genetic variants used in the MR analyses were strongly associated with height (an average F-statistic of 109 per genetic variant) [5].

## Supplementary references

1. Malik R, Chauhan G, Traylor M, Sargurupremraj M, Okada Y, Mishra A, et al. Multiancestry genome-wide association study of 520,000 subjects identifies 32 loci associated with stroke and stroke subtypes. Nat Genet. 2018;50: 524–537. doi:10.1038/s41588-018-0058-3

2. Morris AP. Transethnic Meta-Analysis of Genomewide Association Studies. Genet Epidemiol. 2011;35: 809–822. doi:10.1002/gepi.20630

3. Burgess S, Thompson SG. Mendelian Randomization: Methods for Using Genetic Variants in Causal Estimation. 1st ed. Boca Raton: CRC Press; 2015.

4. Yengo L, Sidorenko J, Kemper KE, Zheng Z, Wood AR, Weedon MN, et al. Meta-analysis of genome-wide association studies for height and body mass index in ∼700000 individuals of European ancestry. Hum Mol Genet. 2018;27: 3641–3649. doi:10.1093/hmg/ddy271

5. Bowden J, Del Greco M F, Minelli C, Davey Smith G, Sheehan NA, Thompson JR. Assessing the suitability of summary data for two-sample Mendelian randomization analyses using MR-Egger regression: the role of the I2 statistic. Int J Epidemiol. 2016;45: 1961–1974. doi:10.1093/ije/dyw220
