# Supplementary material for: Genetic associations of adult height with risk of cardioembolic and other subtypes of ischemic stroke: A mendelian randomization study in multiple ancestries
Source: PLoS Med. 2022 Apr 22;19(4):e1003967. doi: 10.1371/journal.pmed.1003967 (PMC9032370; doi:10.1371/journal.pmed.1003967)
Supplement: S6 Methods — (DOCX) [file pmed.1003967.s009.docx]

## S6 Methods. Sensitivity analyses in MEGASTROKE.

Horizontal pleiotropy may occur if the genetic instrument for height has an effect on ischaemic stroke (or ischaemic stroke subtypes) independent of its effect on measured height, which violates MR assumptions and can lead to inaccurate causal estimates [1]. Directional pleiotropy occurs when horizontal pleiotropy skews causal estimates in a specific direction [2]. The MR-Egger method provides a test of directional pleiotropy (the MR-Egger intercept test) and an estimate of the causal effect (after correction for directional pleiotropy) [2]. The MR-Egger method uses the assumption that the association of each SNP with the exposure is independent of the pleiotropic effect of the SNP (known as the instrument strength independent of direct effect assumption), to provide a valid test of association and a consistent causal effect estimate even if all the SNPs are invalid instrumental variables [2]. The MR pleiotropy residual sum and outlier (MR-PRESSO) method provides a causal estimate before and after correction for horizontal pleiotropy by outlier identification and removal [1]. The weighted median method gives a consistent estimate of the causal effect when at least 50% of the weight of the genetic instrument comes from valid instrumental variables [3,4]. The weighted median, MR-Egger, and MR-PRESSO methods were implemented in R (version 3.3.3), using the MendelianRandomization and MRPRESSO packages. These sensitivity analyses were repeated using effect sizes on height estimated in UKB to test the robustness of the causal estimates to sample overlap between MEGASTROKE and GIANT (2018) [5]. Specifically, 5 studies partly or completed overlapped, comprising about 17% of ischemic stroke cases and about 66% of controls in MEGASTROKE. Sensitivity analyses were not performed in UKB and CKB due to the limited number of stroke subtype cases. As a further sensitivity analysis against pleiotropy we excluded SNPs associated with age at completion of full-time education, diabetes, atrial fibrillation, hypertension, systolic blood pressure, diastolic blood pressure, LDL cholesterol, HDL cholesterol, triglycerides or apolipoprotein B at p<0.001 in the pan-ancestry UK Biobank genome-wide analyses, based on 294 072 to 421 391 participants [6]. (The more stringent p-value of 0.001 rather than p<0.05 [often used in the literature] was regarded as appropriate given the large size of pan-ancestry UK Biobank, since the number of SNPs excluded will increase with the size of the look-up resource.)

## Supplementary references

1. Verbanck M, Chen C-Y, Neale B, Do R. Detection of widespread horizontal pleiotropy in causal relationships inferred from Mendelian randomization between complex traits and diseases. Nat Genet. 2018;50: 693–698. doi:10.1038/s41588-018-0099-7

2. Bowden J, Davey Smith G, Burgess S. Mendelian randomization with invalid instruments: effect estimation and bias detection through Egger regression. Int J Epidemiol. 2015;44: 512–525. doi:10.1093/ije/dyv080

3. Burgess S, Small DS, Thompson SG. A review of instrumental variable estimators for Mendelian randomization. Stat Methods Med Res. 2017;26: 2333–2355. doi:10.1177/0962280215597579

4. Burgess S, Thompson SG. Mendelian Randomization: Methods for Using Genetic Variants in Causal Estimation. 1st ed. Boca Raton: CRC Press; 2015.

5. Yengo L, Sidorenko J, Kemper KE, Zheng Z, Wood AR, Weedon MN, et al. Meta-analysis of genome-wide association studies for height and body mass index in ∼700000 individuals of European ancestry. Hum Mol Genet. 2018;27: 3641–3649. doi:10.1093/hmg/ddy271

6. Pan-ancestry UK Biobank (Pan-UKBB). [cited 16 Dec 2021]. Available: https://pan.ukbb.broadinstitute.org/
