# Supplementary material for: Genetic associations of adult height with risk of cardioembolic and other subtypes of ischemic stroke: A mendelian randomization study in multiple ancestries
Source: PLoS Med. 2022 Apr 22;19(4):e1003967. doi: 10.1371/journal.pmed.1003967 (PMC9032370; doi:10.1371/journal.pmed.1003967)
Supplement: S7 Methods — CKB, China Kadoorie Biobank; UKB, UK Biobank. (DOCX) [file pmed.1003967.s010.docx]

## S7 Methods. Further details of observational analyses in UK Biobank and China Kadoorie Biobank.

In UKB, observational analyses were among participants with no prior history of myocardial infarction (algorithmically-defined myocardial infarction occurring prior to enrolment) or stroke (algorithmically-defined stroke of any type prior to enrolment). In CKB, observational analyses were among participants with no prior history of ischaemic heart disease (self-reported at enrolment) or stroke (stroke of any type self-reported at enrolment). Hazard ratios for the associations of measured height with incident ischaemic stroke and ischaemic stroke subtypes, were estimated by Cox regression stratified by age-at-risk (in 5-year groups), sex, and region (10 regions in CKB), with adjustment for additional potential confounders including year of birth (as a continuous variable), smoking status (never smoker, ex-smoker, and current smoker), number of cigarettes smoked (daily), systolic blood pressure, diastolic blood pressure, diagnosed hypertension, diagnosed diabetes, self-rated walking pace (UKB only), and level of education (primary/no qualification/unknown, secondary, tertiary).
