# Supplementary material for: Genetic associations of adult height with risk of cardioembolic and other subtypes of ischemic stroke: A mendelian randomization study in multiple ancestries
Source: PLoS Med. 2022 Apr 22;19(4):e1003967. doi: 10.1371/journal.pmed.1003967 (PMC9032370; doi:10.1371/journal.pmed.1003967)
Supplement: S1 Table — Data are n (%) or mean (SD) unless otherwise stated. In UKB, blood lipids measurements were available in 85% to 93% of participants, lung function in 71%, and anthropometric traits ≥98% (S1 Fig). In CKB, blood lipids measurements were available in 4% of participants and lung function in 87% (S2 Fig). BMI, body mass index; CKB, China Kadoorie Biobank; FEV1, forced expiratory volume in 1 second; FVC, forced vital capacity; HDL, high-density lipoprotein; IQR, interquartile range; LDL, low-density lipoprotein; NA, not available; UKB, UK Biobank. (DOCX) [file pmed.1003967.s011.docx]

## S1 Table. Baseline characteristics of included UK Biobank and China Kadoorie Biobank participants without prior major cardiovascular disease.

| **Baseline characteristic** | **UK Biobank (n=482928)** | | | **China Kadoorie Biobank (n=490067)** | | |
| --- | --- | --- | --- | --- | --- | --- |
| Age groups |  | |  | |  | |
| <40 years | 6 | (0.0%) | | 77763 | | (15.9%) |
| 40–49 years | 115791 | (24.0%) | | 150726 | | (30.8%) |
| 50–59 years | 162025 | (33.6%) | | 150672 | | (30.7%) |
| 60–69 years | 202899 | (42.0%) | | 82283 | | (16.8%) |
| ≥70 years | 2207 | (0.5%) | | 28623 | | (5.8%) |
| Age (years) | 56.4 | (8.1) | | 51.6 | | (10.6) |
| Year of birth (year), median (IQR) | 1951 | (13.0) | | 1955 | | (16.0) |
| Women | 267166 | (55.3%) | | 289703 | | (59.1%) |
| Highest attained education |  |  | |  | |  |
| Primary/no qualification/unknown | 87959 | (18.2%) | | 249057 | | (50.8%) |
| Secondary | 212846 | (44.1%) | | 213097 | | (43.5%) |
| Tertiary | 182123 | (37.7%) | | 27913 | | (5.7%) |
| Current smoker, men | 26609 | (12.3%) | | 124170 | | (62.0%) |
| Current smoker, women | 23576 | (8.8%) | | 6626 | | (2.3%) |
| Diagnosed diabetes | 32877 | (6.8%) | | 26422 | | (5.4%) |
| Diagnosed atrial fibrillation | 7040 | (1.5%) | | NA | |  |
| Diagnosed hypertension | 154364 | (32.0%) | | 48568 | | (9.9%) |
| Systolic blood pressure groups | | | | | | |
| <120 mmHg | 78722 | (16.3%) | | 159478 | | (32.5%) |
| 120–140 mmHg | 198298 | (41.1%) | | 193671 | | (39.5%) |
| 140–160 mmHg | 146412 | (30.3%) | | 90222 | | (18.4%) |
| ≥160 mmHg | 59496 | (12.3%) | | 46696 | | (9.5%) |
| Systolic blood pressure (mmHg) | 137.9 | (18.6) | | 130.6 | | (21.1) |
| Diastolic blood pressure (mmHg) | 82.3 | (10.1) | | 77.6 | | (11.1) |
| LDL cholesterol (mmol/L) | 3.6 | (0.9) | | 2.4 | | (0.7) |
| HDL cholesterol (mmol/L) | 1.5 | (0.4) | | 1.2 | | (0.3) |
| Triglycerides (mmol/L) | 1.7 | (1.0) | | 2.0 | | (1.6) |
| Apolipoprotein B (g/L) | 1.0 | (0.2) | | 0.8 | | (0.2) |
| Lung function measures within sex | | | | | | |
| FEV1, men (L) | 3.4 | (0.7) | | 2.7 | | (0.7) |
| FEV1, women (L) | 2.4 | (0.5) | | 2.0 | | (0.5) |
| FVC, men (L) | 4.5 | (0.9) | | 3.2 | | (0.8) |
| FVC, women (L) | 3.2 | (0.6) | | 2.4 | | (0.5) |
| Anthropometric traits within sex | | | | | | |
| BMI, men (kg/m^2^) | 27.8 | (4.2) | | 23.4 | | (3.2) |
| BMI, women (kg/m^2^) | 27.0 | (5.1) | | 23.7 | | (3.4) |
| Waist-to-hip, men (ratio) | 0.9 | (0.1) | | 0.9 | | (0.1) |
| Waist-to-hip, women (ratio) | 0.8 | (0.1) | | 0.9 | | (0.1) |
| Weight, men (kg) | 85.8 | (14.3) | | 64.0 | | (10.9) |
| Weight, women (kg) | 71.4 | (14.0) | | 56.5 | | (9.4) |
| Lean body mass, men (kg) | 63.7 | (7.8) | | 49.5 | | (6.1) |
| Lean body mass, women (kg) | 44.5 | (5.0) | | 38.0 | | (4.1) |
| Height, men (cm) | 175.7 | (6.8) | | 165.2 | | (6.5) |
| Height, women (cm) | 162.5 | (6.3) | | 154.2 | | (6.0) |
