# Supplementary material for: Genetic associations of adult height with risk of cardioembolic and other subtypes of ischemic stroke: A mendelian randomization study in multiple ancestries
Source: PLoS Med. 2022 Apr 22;19(4):e1003967. doi: 10.1371/journal.pmed.1003967 (PMC9032370; doi:10.1371/journal.pmed.1003967)
Supplement: S2 Table — Ancestry was predominantly self-reported in the 29 genome-wide studies comprising the MEGASTROKE consortium data. *Other ancestry included Latin American and mixed Asian ancestry. (DOCX) [file pmed.1003967.s012.docx]

## S2 Table. Ancestry composition of stroke cases in MEGASTROKE.

|  | **Number of cases (%)** | | | | | | | |
| --- | --- | --- | --- | --- | --- | --- | --- | --- |
| **Ancestry** | **Ischaemic stroke** | | **Cardioembolic stroke** | | **Large-artery stroke** | | **Small-vessel stroke** | |
| European | 34217 | (57%) | 7193 | (80%) | 4373 | (65%) | 5386 | (46%) |
| East Asian | 17369 | (29%) | 847 | (9%) | 1626 | (24%) | 5096 | (44%) |
| African | 5541 | (9%) | 414 | (5%) | 259 | (4%) | 694 | (6%) |
| South Asian | 2437 | (4%) | 422 | (5%) | 355 | (5%) | 314 | (3%) |
| Other ancestry* | 777 | (1%) | 130 | (1%) | 75 | (1%) | 220 | (2%) |
| Total | 60341 | (100%) | 9006 | (100%) | 6688 | (100%) | 11710 | (100%) |
