# Supplementary material for: Genetic associations of adult height with risk of cardioembolic and other subtypes of ischemic stroke: A mendelian randomization study in multiple ancestries
Source: PLoS Med. 2022 Apr 22;19(4):e1003967. doi: 10.1371/journal.pmed.1003967 (PMC9032370; doi:10.1371/journal.pmed.1003967)
Supplement: S3 Table — *Each individual genetic instrument for height, based on GIANT or Biobank Japan SNPs, was linkage disequilibrium pruned (r2 < 0.05). †The number of linkage disequilibrium pruned SNPs available in UKB or CKB. ‡Beta estimate of measured height regressed on the genetic risk score for height, adjusted for age, age2, sex, region (in CKB only), genomic principal components, and genotyping array type. Biobank Japan, Biobank Japan genome-wide association study (2019) [20]; CKB, China Kadoorie Biobank; GIANT (2014), Genetic Investigation of Anthropometric Traits (2014) [19]; GIANT (2018), Genetic Investigation of Anthropometric Traits (2018) [18]; R2, the proportion of the residual variance of height explained by the genetic risk score for height (the coefficient of determination); SNP, single nucleotide polymorphism; UKB, UK Biobank. (DOCX) [file pmed.1003967.s013.docx]

## S3 Table. Percentage of height variance explained (R^2^) by genetic instruments for height in UK Biobank and China Kadoorie Biobank.

|  | **Genetic risk score** | | |
| --- | --- | --- | --- |
| **Genetic instrument for height*** | **No. of SNPs included†** | **R^2^** | **Beta‡** |
| **UK Biobank (n=336750)** |  |  |  |
| GIANT (2014) SNPs with single-variant effect sizes | 641 | 17.03% | 0.91 |
| **China Kadoorie Biobank random sample (n=53346)** |  |  |  |
| GIANT (2018) SNPs with single-variant effect sizes | 2337 | 11.44% | 0.71 |
| Biobank Japan SNPs with single-variant effect sizes | 517 | 11.02% | 0.86 |
| GIANT (2018) and Biobank Japan SNPs with single-variant effect sizes (equal weighting) | 2854 | 15.24% | 1.05 |
| GIANT (2018) and Biobank Japan SNPs with single-variant effect sizes (25% and 75%, respectively) | 2854 | 14.27% | 1.04 |
| GIANT (2018) and Biobank Japan SNPs with single-variant effect sizes (75% and 25%, respectively) | 2854 | 13.79% | 0.90 |
