# Supplementary material for: Genetic associations of adult height with risk of cardioembolic and other subtypes of ischemic stroke: A mendelian randomization study in multiple ancestries
Source: PLoS Med. 2022 Apr 22;19(4):e1003967. doi: 10.1371/journal.pmed.1003967 (PMC9032370; doi:10.1371/journal.pmed.1003967)
Supplement: S4 Table — CKB, China Kadoorie Biobank; UKB, UK Biobank. (DOCX) [file pmed.1003967.s014.docx]

## S4 Table. Associations of the genetic risk score for height with ischaemic stroke and its subtypes in UK Biobank and China Kadoorie Biobank.

|  | **No. of events** | **Association statistics** | | |
| --- | --- | --- | --- | --- |
| **Study and ischaemic stroke subtype** |  | **Beta (log odds ratio)** | **Standard error** | **P-value** |
| **UK Biobank** |  |  |  |  |
| Presumed cardioembolic stroke | 454 | 0.07 | 0.12 | 0.53 |
| Other ischaemic stroke subtypes | 3601 | -0.03 | 0.04 | 0.49 |
| All ischaemic stroke | 4055 | -0.02 | 0.04 | 0.66 |
| **China Kadoorie Biobank** |  |  |  |  |
| Presumed cardioembolic stroke | 133 | 0.18 | 0.22 | 0.43 |
| Other non-lacunar stroke | 2205 | -0.11 | 0.06 | 0.06 |
| Lacunar stroke | 2138 | -0.01 | 0.06 | 0.85 |
| All ischaemic stroke | 10297 | -0.06 | 0.03 | 0.05 |
