# Supplementary material for: Genetic associations of adult height with risk of cardioembolic and other subtypes of ischemic stroke: A mendelian randomization study in multiple ancestries
Source: PLoS Med. 2022 Apr 22;19(4):e1003967. doi: 10.1371/journal.pmed.1003967 (PMC9032370; doi:10.1371/journal.pmed.1003967)
Supplement: S5 Table — *OR per 1 SD genetically determined taller height. The numbers of events reported for MEGASTROKE were the maximum number of cases available in the genetic summary data. GIANT (2018), Genetic Investigation of Anthropometric Traits (2018) [18]; IVW, inverse variance weighted; MR, mendelian randomization; MR–PRESSO, Mendelian Randomization Pleiotropy RESidual Sum and Outlier; OR, odds ratio; SD, standard deviation. (DOCX) [file pmed.1003967.s015.docx]

## S5 Table (page 1 of 2). Weighted median, MR-Egger, and MR-PRESSO sensitivity analyses of the associations of genetically-determined height with ischaemic stroke and its subtypes in MEGASTROKE.

| **Source of SNP effect sizes on height, ancestry subset, and ischaemic stroke subtype** | | **No. of events** | **IVW random-effects meta-analysis** | |  | **Weighted median** | |
| --- | --- | --- | --- | --- | --- | --- | --- |
|  |  |  | **OR (95% CI)*** | **P-value** |  | **OR (95% CI)*** | **P-value** |
| **GIANT (2018) SNP effect sizes on height** | | | | |  |  |  |
| **Multiple ancestry** | |  |  |  |  |  |  |
|  | Cardioembolic stroke | 9006 | 1.13 (1.07, 1.19) | <0.001 |  | 1.23 (1.13, 1.34) | <0.001 |
|  | Large-artery stroke | 6688 | 0.89 (0.84, 0.95) | <0.001 |  | 0.90 (0.81, 0.99) | 0.04 |
|  | Small-vessel stroke | 11710 | 0.87 (0.83, 0.92) | <0.001 |  | 0.86 (0.79, 0.94) | <0.001 |
|  | All ischaemic stroke | 60341 | 0.96 (0.94, 0.99) | 0.007 |  | 0.98 (0.94, 1.02) | 0.28 |
| **European ancestry** | |  |  |  |  |  |  |
|  | Cardioembolic stroke | 7193 | 1.14 (1.08, 1.21) | <0.001 |  | 1.19 (1.08, 1.30) | <0.001 |
|  | Large-artery stroke | 4373 | 0.88 (0.82, 0.95) | 0.001 |  | 0.87 (0.77, 0.98) | 0.03 |
|  | Small-vessel stroke | 5386 | 0.85 (0.80, 0.91) | <0.001 |  | 0.84 (0.75, 0.94) | 0.002 |
|  | All ischaemic stroke | 34217 | 0.96 (0.93, 0.99) | 0.02 |  | 0.96 (0.92, 1.01) | 0.15 |
| **UK Biobank estimated SNP effect sizes on height** | | | | |  |  |  |
| **Multiple ancestry** | |  |  |  |  |  |  |
|  | Cardioembolic stroke | 9006 | 1.12 (1.07, 1.18) | <0.001 |  | 1.23 (1.13, 1.33) | <0.001 |
|  | Large-artery stroke | 6688 | 0.88 (0.83, 0.93) | <0.001 |  | 0.89 (0.81, 0.97) | 0.01 |
|  | Small-vessel stroke | 11710 | 0.90 (0.86, 0.95) | <0.001 |  | 0.90 (0.83, 0.97) | 0.007 |
|  | All ischaemic stroke | 60341 | 0.96 (0.94, 0.99) | 0.005 |  | 0.98 (0.94, 1.02) | 0.35 |
| **European ancestry** | |  |  |  |  |  |  |
|  | Cardioembolic stroke | 7193 | 1.13 (1.07, 1.19) | <0.001 |  | 1.17 (1.07, 1.27) | <0.001 |
|  | Large-artery stroke | 4373 | 0.88 (0.82, 0.94) | <0.001 |  | 0.85 (0.76, 0.95) | 0.005 |
|  | Small-vessel stroke | 5386 | 0.87 (0.82, 0.93) | <0.001 |  | 0.87 (0.79, 0.97) | 0.01 |
|  | All ischaemic stroke | 34217 | 0.96 (0.93, 0.99) | 0.007 |  | 0.97 (0.92, 1.01) | 0.13 |

## S5 Table (page 2 of 2). Weighted median, MR-Egger, and MR-PRESSO sensitivity analyses of the associations of genetically-determined height with ischaemic stroke and its subtypes in MEGASTROKE.

| **Source of SNP effect sizes on height, ancestry subset, and ischaemic stroke subtype** | | **No. of events** | **MR-Egger** | | |  | **MR-PRESSO** | | |
| --- | --- | --- | --- | --- | --- | --- | --- | --- | --- |
|  |  |  | **OR (95% CI)*** | **P-value of OR** | **P-value of intercept** |  | **No. of outlying variants** | **Outlier corrected OR (95% CI)*** | **P-value** |
| **GIANT (2018) SNP effect sizes on height** | | | |  |  |  |  |  |  |
| **Multiple ancestry** | |  |  |  |  |  |  |  |  |
|  | Cardioembolic stroke | 9006 | 1.18 (1.07, 1.31) | 0.002 | 0.32 |  | 1 | 1.13 (1.08, 1.20) | <0.001 |
|  | Large-artery stroke | 6688 | 0.88 (0.77, 0.99) | 0.04 | 0.72 |  | 2 | 0.90 (0.84, 0.95) | <0.001 |
|  | Small-vessel stroke | 11710 | 0.81 (0.73, 0.90) | <0.001 | 0.08 |  | 2 | 0.88 (0.83, 0.92) | <0.001 |
|  | All ischaemic stroke | 60341 | 0.97 (0.91, 1.02) | 0.21 | 0.90 |  | 4 | 0.97 (0.94, 1.00) | 0.03 |
| **European ancestry** | |  |  |  |  |  |  |  |  |
|  | Cardioembolic stroke | 7193 | 1.18 (1.05, 1.33) | 0.005 | 0.49 |  | 1 | 1.14 (1.08, 1.21) | <0.001 |
|  | Large-artery stroke | 4373 | 0.89 (0.76, 1.04) | 0.14 | 0.89 |  | 0 | 0.88 (0.82, 0.95) | NA |
|  | Small-vessel stroke | 5386 | 0.81 (0.71, 0.93) | 0.002 | 0.39 |  | 0 | 0.85 (0.80, 0.91) | NA |
|  | All ischaemic stroke | 34217 | 0.98 (0.92, 1.04) | 0.46 | 0.59 |  | 3 | 0.97 (0.94, 1.00) | 0.03 |
| **UK Biobank estimated SNP effect sizes on height** | | | |  |  |  |  |  |  |
| **Multiple ancestry** | |  |  |  |  |  |  |  |  |
|  | Cardioembolic stroke | 9006 | 1.16 (1.05, 1.27) | 0.002 | 0.46 |  | 1 | 1.13 (1.07, 1.18) | <0.001 |
|  | Large-artery stroke | 6688 | 0.86 (0.77, 0.96) | 0.008 | 0.62 |  | 1 | 0.88 (0.83, 0.93) | <0.001 |
|  | Small-vessel stroke | 11710 | 0.85 (0.77, 0.93) | <0.001 | 0.14 |  | 2 | 0.90 (0.86, 0.95) | <0.001 |
|  | All ischaemic stroke | 60341 | 0.96 (0.91, 1.01) | 0.10 | 0.85 |  | 5 | 0.97 (0.95, 1.00) | 0.03 |
| **European ancestry** | |  |  |  |  |  |  |  |  |
|  | Cardioembolic stroke | 7193 | 1.16 (1.04, 1.29) | 0.006 | 0.59 |  | 0 | 1.13 (1.07, 1.19) | NA |
|  | Large-artery stroke | 4373 | 0.87 (0.76, 1.00) | 0.05 | 0.93 |  | 1 | 0.88 (0.82, 0.94) | <0.001 |
|  | Small-vessel stroke | 5386 | 0.85 (0.76, 0.97) | 0.01 | 0.66 |  | 1 | 0.88 (0.82, 0.93) | <0.001 |
|  | All ischaemic stroke | 34217 | 0.97 (0.91, 1.02) | 0.22 | 0.84 |  | 3 | 0.96 (0.94, 0.99) | 0.02 |
