# Supplementary material for: Genetic associations of adult height with risk of cardioembolic and other subtypes of ischemic stroke: A mendelian randomization study in multiple ancestries
Source: PLoS Med. 2022 Apr 22;19(4):e1003967. doi: 10.1371/journal.pmed.1003967 (PMC9032370; doi:10.1371/journal.pmed.1003967)
Supplement: S6 Table — *The number of SNPs available for each stroke subtype varied from 2,084 to 2,277 in the main genetic instrument for height, from 1,377 to 1,514 in the restricted genetic instrument in (A) and from 1,114 to 1,180 in the genetic instrument in (B). See S4 Methods (further details of instruments for genetically determined height). †In the pan-ancestry genetic analysis of the UKB (Pan-UKBB) based on 294,072 to 421,391 participants [26]. Percentages of SNPs associated (at p < 0.001) with each risk factor were age at completion of full-time education (2.8%), diabetes (2.2%), atrial fibrillation (1.4%), hypertension (7.1%), systolic blood pressure (10.3%), diastolic blood pressure (8.9%), LDL cholesterol (5.7%), HDL cholesterol (10.7%), triglycerides (10.5%), and apolipoprotein B (6.5%). HDL, high-density lipoprotein; LD, linkage disequilibrium; LDL, low-density lipoprotein; OR, odds ratio; SNP, single nucleotide polymorphism; UKB, UK Biobank. (DOCX) [file pmed.1003967.s016.docx]

## S6 Table. Additional sensitivity analyses of the associations of genetically-determined height with ischaemic stroke and its subtypes in MEGASTROKE using (A) a restricted genetic instrument excluding SNPs associated with age at completion of full-time education, diabetes, atrial fibrillation, hypertension, systolic blood pressure, diastolic blood pressure, LDL cholesterol, HDL cholesterol, triglycerides or apolipoprotein B, and (B) a genetic instrument for height with a stricter level of LD pruning (r^2^<0.001).

|  |  |  | **Main genetic instrument  for height including SNPs  LD pruned at r^2^<0.05** | |  | 1. **Restricted genetic instrument  for height excluding SNPs  associated at p<0.001 with  potential pleiotropic risk factors**† | |  | 1. **Genetic instrument  for height with stricter  LD pruning at r^2^<0.001** | |
| --- | --- | --- | --- | --- | --- | --- | --- | --- | --- | --- |
|  |  |  |  |  |  |  |  |  |  |  |
| **No. of SNPs included*** |  |  | **2278** | |  | **1515** | |  | **1180** | |
| **Ancestry subset and ischaemic stroke subtype** | **No. of events** |  | **OR (95% CI)** | **P-value** |  | **OR (95% CI)** | **P-value** |  | **OR (95% CI)** | **P-value** |
| **Multiple ancestry** |  |  |  |  |  |  |  |  |  |  |
| Cardioembolic stroke | 9006 |  | 1.13 (1.07, 1.19) | <0.001 |  | 1.19 (1.11, 1.27) | <0.001 |  | 1.14 (1.07, 1.21) | <0.001 |
| Large-artery stroke | 6688 |  | 0.89 (0.84, 0.95) | <0.001 |  | 0.89 (0.82, 0.97) | 0.006 |  | 0.91 (0.84, 0.98) | 0.01 |
| Small-vessel stroke | 11710 |  | 0.87 (0.83, 0.92) | <0.001 |  | 0.93 (0.87, 1.00) | 0.04 |  | 0.88 (0.82, 0.94) | <0.001 |
| All ischaemic stroke | 60341 |  | 0.96 (0.94, 0.99) | 0.007 |  | 0.98 (0.95, 1.02) | 0.32 |  | 0.97 (0.94, 1.00) | 0.07 |
| **European ancestry** |  |  |  |  |  |  |  |  |  |  |
| Cardioembolic stroke | 7193 |  | 1.14 (1.08, 1.21) | <0.001 |  | 1.22 (1.12, 1.31) | <0.001 |  | 1.14 (1.06, 1.22) | <0.001 |
| Large-artery stroke | 4373 |  | 0.88 (0.82, 0.95) | 0.001 |  | 0.90 (0.81, 0.99) | 0.04 |  | 0.90 (0.82, 0.99) | 0.03 |
| Small-vessel stroke | 5386 |  | 0.85 (0.80, 0.91) | <0.001 |  | 0.92 (0.84, 1.01) | 0.08 |  | 0.85 (0.78, 0.93) | <0.001 |
| All ischaemic stroke | 34217 |  | 0.96 (0.93, 0.99) | 0.02 |  | 0.99 (0.95, 1.04) | 0.78 |  | 0.97 (0.93, 1.01) | 0.17 |
