# Supplementary material for: Genetic associations of adult height with risk of cardioembolic and other subtypes of ischemic stroke: A mendelian randomization study in multiple ancestries
Source: PLoS Med. 2022 Apr 22;19(4):e1003967. doi: 10.1371/journal.pmed.1003967 (PMC9032370; doi:10.1371/journal.pmed.1003967)
Supplement: S7 Table — Each individual genetic instrument for height, based on GIANT or Biobank Japan SNPs, was linkage disequilibrium pruned (r2 < 0.05). The category “All ischemic stroke” includes additional unsubtyped ischemic strokes. Genetic associations in CKB were adjusted for age, age2, sex, region, genomic principal components, and genotyping array type. Biobank Japan, Biobank Japan genome-wide association study (2019) [20]; CKB, China Kadoorie Biobank; GIANT (2018), Genetic Investigation of Anthropometric Traits (2018) [18]; OR, odds ratio; R2, the proportion of the residual variance of height explained by the genetic risk score for height (the coefficient of determination); SNP, single nucleotide polymorphism. (DOCX) [file pmed.1003967.s017.docx]

## S7 Table. Associations of genetically-determined height with ischaemic stroke and its subtypes in China Kadoorie Biobank shown for different genetic instruments.

|  |  |  | **GIANT (2018) instrument including 2337 SNPs** | |  | **Biobank Japan instrument including 517SNPs** | |  | **Average of the GIANT and Biobank Japan instruments** | |
| --- | --- | --- | --- | --- | --- | --- | --- | --- | --- | --- |
| **R^2^** |  |  | **11.44%** | |  | **11.02%** | |  | **15.24%** | |
| **Ischaemic stroke subtypes** | **No. of events** |  | **OR (95% CI)** | **P-value** |  | **OR (95% CI)** | **P-value** |  | **OR (95% CI)** | **P-value** |
| Presumed cardioembolic stroke | 133 |  | 1.31 (0.79, 2.18) | 0.29 |  | 1.06 (0.63, 1.78) | 0.82 |  | 1.20 (0.77, 1.85) | 0.43 |
| Other non-lacunar stroke | 2205 |  | 0.90 (0.79, 1.03) | 0.12 |  | 0.89 (0.77, 1.02) | 0.09 |  | 0.89 (0.80, 1.00) | 0.06 |
| Lacunar stroke | 2138 |  | 0.95 (0.83, 1.10) | 0.52 |  | 1.03 (0.90, 1.19) | 0.65 |  | 0.99 (0.88, 1.12) | 0.85 |
| All ischaemic stroke | 10297 |  | 0.93 (0.86, 1.00) | 0.06 |  | 0.95 (0.88, 1.02) | 0.16 |  | 0.94 (0.88, 1.00) | 0.05 |
