# Supplementary material for: Genetic associations of adult height with risk of cardioembolic and other subtypes of ischemic stroke: A mendelian randomization study in multiple ancestries
Source: PLoS Med. 2022 Apr 22;19(4):e1003967. doi: 10.1371/journal.pmed.1003967 (PMC9032370; doi:10.1371/journal.pmed.1003967)
Supplement: S8 Table — For UKB and CKB, respectively, the SDs of directly measured height were 6.8 cm versus 6.5 cm for men and 6.3 cm versus 6.0 cm for women. *Associations were stratified by age at risk (in 5-year groups), sex, and region (in CKB only) and adjusted for year of birth. †Additional potential confounders included year of birth, smoking status, number of cigarettes smoked, systolic blood pressure, diastolic blood pressure, diagnosed hypertension, diagnosed diabetes, self-rated walking pace (UKB only), and level of education (S6 Methods). CKB, China Kadoorie Biobank; HR, hazard ratio; SD, standard deviation; UKB, UK Biobank. (DOCX) [file pmed.1003967.s018.docx]

## S8 Table. Associations of measured height with ischaemic stroke and its subtypes in UK Biobank and China Kadoorie Biobank.

| **Study and ischaemic stroke subtype** | | **No. of events** | **Observational associations after basic stratification and adjustment*** | |  | **Observational associations after further adjustment for additional potential confounders†** | |
| --- | --- | --- | --- | --- | --- | --- | --- |
|  |  |  | **HR (95% CI)** | **P-value** |  | **HR (95% CI)** | **P-value** |
| **UK Biobank** | |  |  |  |  |  |  |
|  | Presumed cardioembolic stroke | 495 | 1.09 (1.00, 1.19) | 0.06 |  | 1.17 (1.07, 1.28) | <0.001 |
|  | Other ischaemic stroke subtypes | 3203 | 0.89 (0.86, 0.92) | <0.001 |  | 0.96 (0.92, 0.99) | 0.02 |
|  | All ischaemic stroke | 3698 | 0.91 (0.88, 0.94) | <0.001 |  | 0.98 (0.95, 1.02) | 0.33 |
| **China Kadoorie Biobank** | |  |  |  |  |  |  |
|  | Presumed cardioembolic stroke | 410 | 1.10 (1.00, 1.21) | 0.05 |  | 1.09 (0.99, 1.20) | 0.09 |
|  | Other non-lacunar stroke | 7503 | 0.96 (0.93, 0.98) | <0.001 |  | 0.93 (0.91, 0.96) | <0.001 |
|  | Lacunar stroke | 6840 | 0.98 (0.96, 1.00) | 0.11 |  | 0.96 (0.94, 0.99) | 0.002 |
|  | All ischaemic stroke | 37947 | 0.97 (0.96, 0.98) | <0.001 |  | 0.96 (0.95, 0.97) | <0.001 |
