# Supplementary material for: Genetic associations of adult height with risk of cardioembolic and other subtypes of ischemic stroke: A mendelian randomization study in multiple ancestries
Source: PLoS Med. 2022 Apr 22;19(4):e1003967. doi: 10.1371/journal.pmed.1003967 (PMC9032370; doi:10.1371/journal.pmed.1003967)
Supplement: S9 Table — *Effects are the ORs per 1 SD genetically determined taller height, adjusted for age, age2, sex, region (in CKB only), genomic principal components, and genotyping array type. For UKB and CKB, respectively, the SDs of directly measured height were 6.8 cm versus 6.5 cm for men and 6.3 cm versus 6.0 cm for women. †Each pair of signs indicates the direction of the estimated effect for UKB (first sign) and CKB (second sign). CKB, China Kadoorie Biobank; OR, odds ratio; SD, standard deviation; UKB, UK Biobank. (DOCX) [file pmed.1003967.s019.docx]

## S9 Table. Associations of genetically-determined height with other cardiovascular risk factors—smoking status and education.

|  | **UK Biobank (n=336750)** | | |  | **China Kadoorie Biobank (n=53346)** | | | **Directional consistency†** |
| --- | --- | --- | --- | --- | --- | --- | --- | --- |
| **Baseline characteristic** | **Effect (95% CI) per 1-SD genetically-determined taller height*** | **Z-statistic** | **P-value** |  | **Effect (95% CI) per 1-SD genetically-determined taller height*** | **Z-statistic** | **P-value** |  |
| **Smoker status (odds ratio)** | |  |  |  |  |  |  |  |
| Current smoker | 0.98 (0.95, 1.01) | -1.4 | 0.16 |  | 1.03 (0.96, 1.10) | 0.9 | 0.39 | -+ |
| **Education group (odds ratio)** |  |  |  |  |  |  |  |  |
| Tertiary education | 1.10 (1.08, 1.12) | 11.6 | <0.001 |  | 0.99 (0.90, 1.09) | -0.9 | 0.87 | +- |
