# Supplementary figures and images for: Genetic associations of adult height with risk of cardioembolic and other subtypes of ischemic stroke: A mendelian randomization study in multiple ancestries
Source: PLoS Med. 2022 Apr 22;19(4):e1003967. doi: 10.1371/journal.pmed.1003967 (PMC9032370; doi:10.1371/journal.pmed.1003967)

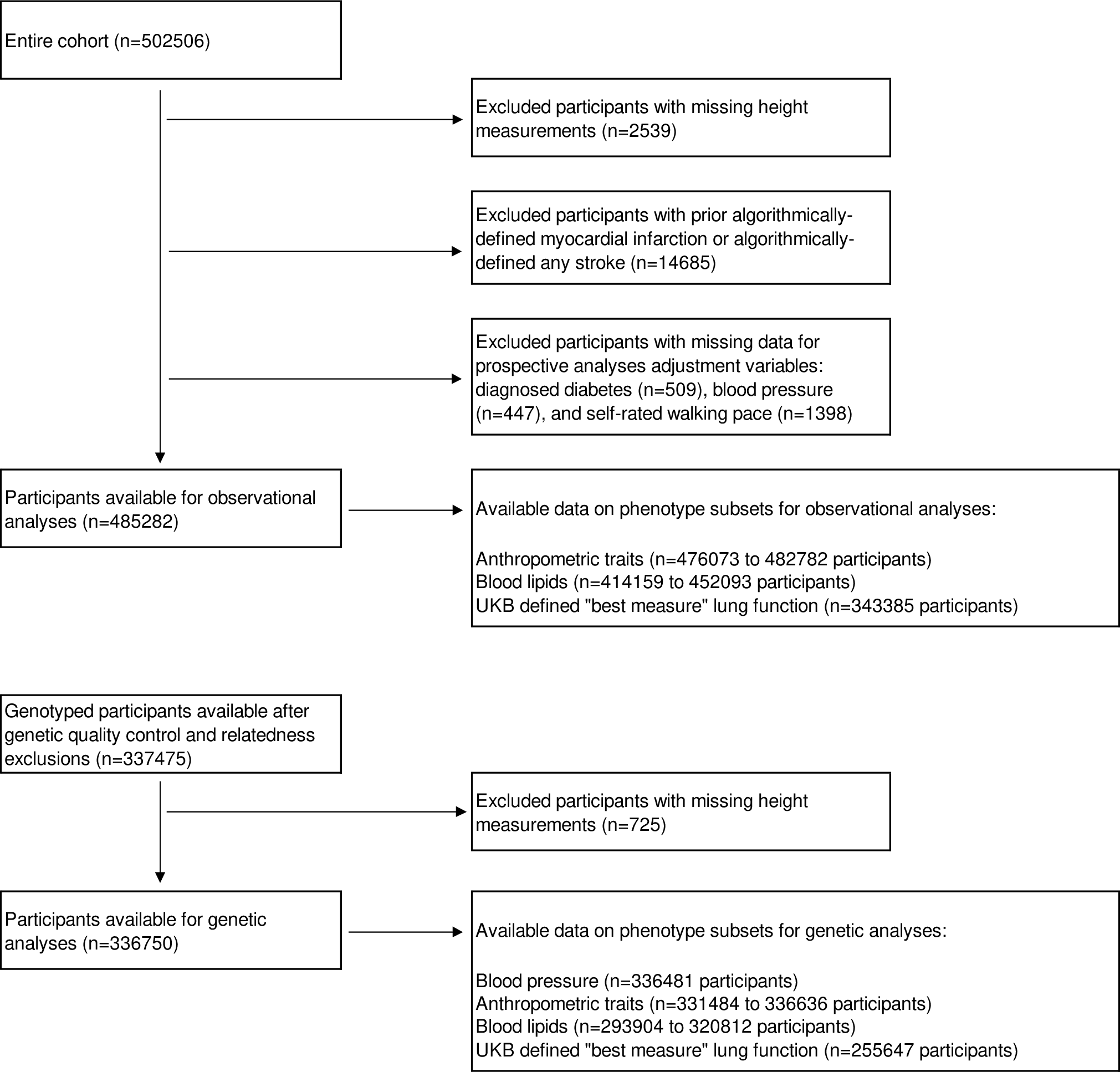

Supplement: S1 Fig — UKB, UK Biobank. (TIF) [file pmed.1003967.s020.tif]

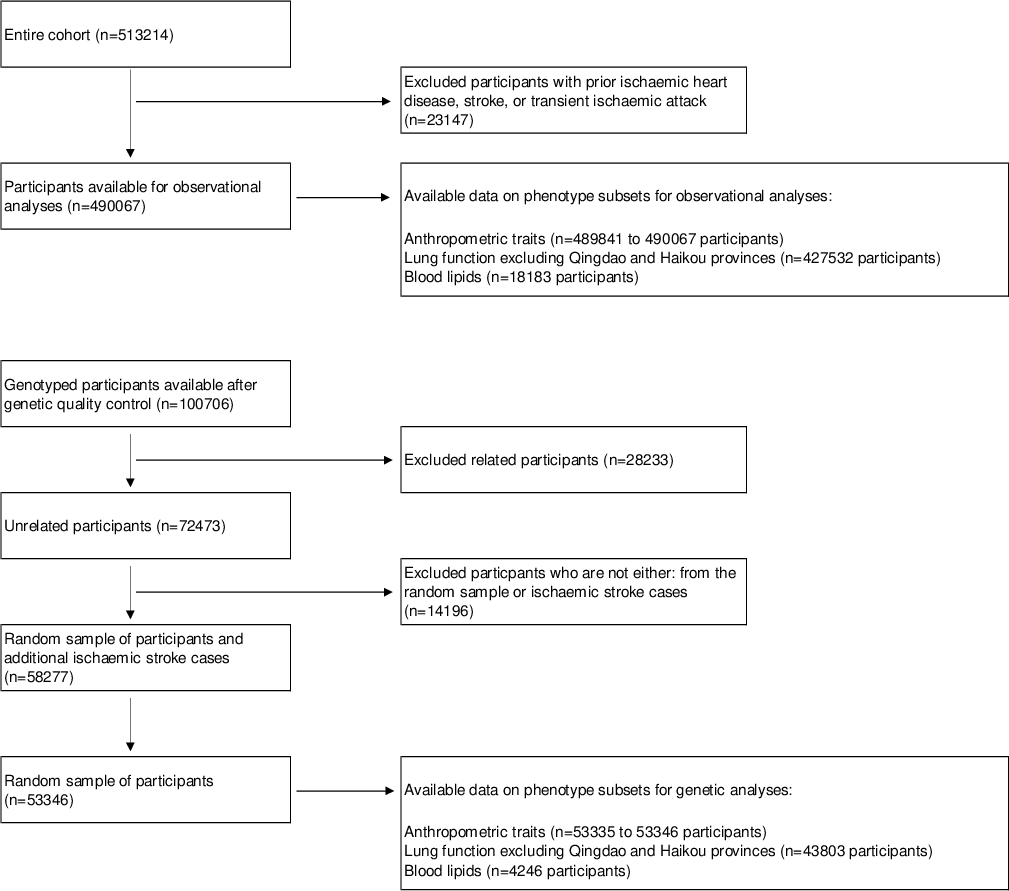

Supplement: S2 Fig — CKB, China Kadoorie Biobank. (TIF) [file pmed.1003967.s021.tif]

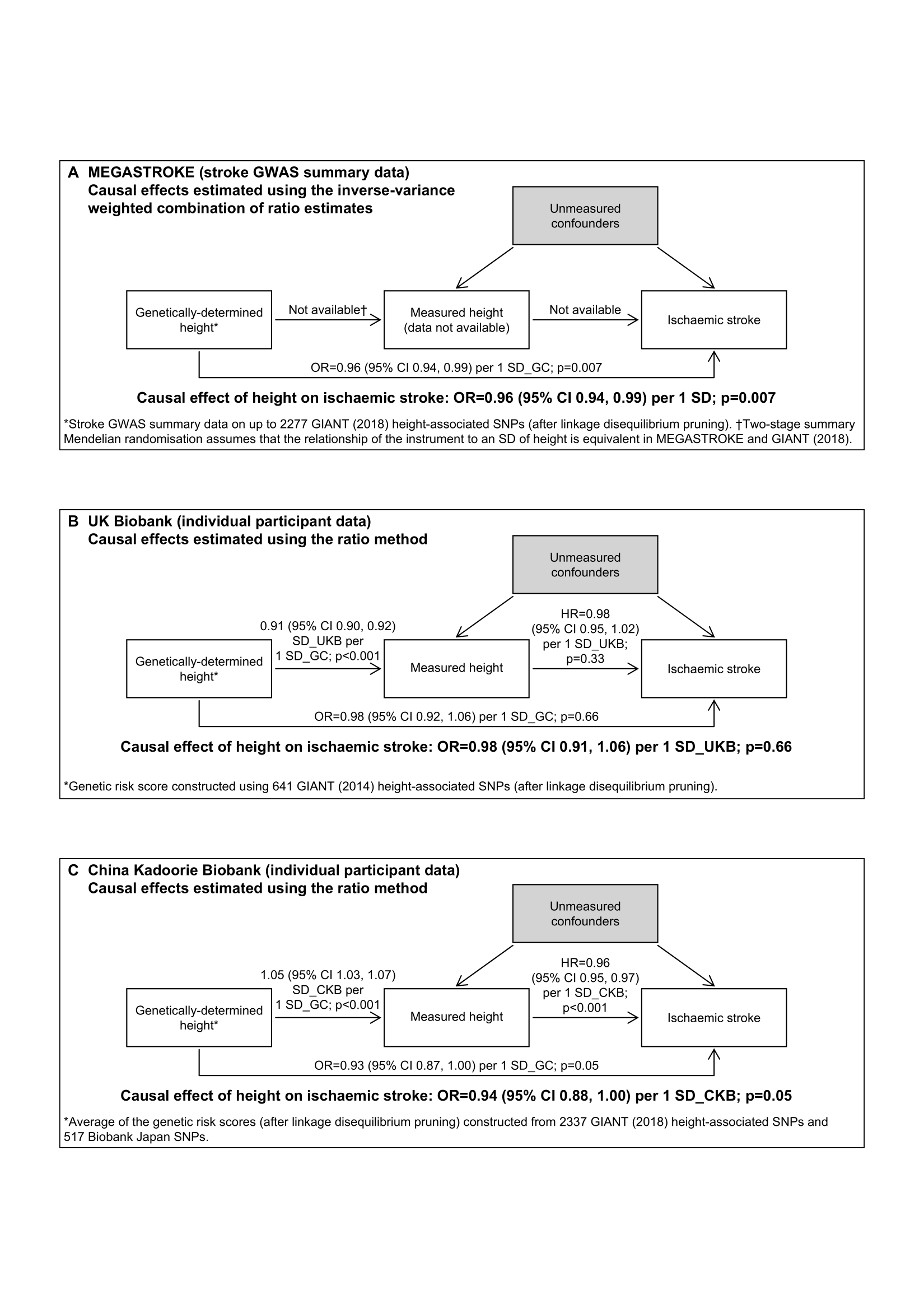

Supplement: S3 Fig — Biobank Japan, Biobank Japan genome-wide association study (2019) [20]; CKB, China Kadoorie Biobank; GC, genetic consortia (which differs between studies); GIANT (2014), Genetic Investigation of Anthropometric Traits (2014) [19]; GIANT (2018), Genetic Investigation of Anthropometric Traits (2018) [18]; GWAS, genome-wide association study; HR, hazard ratio; MR, mendelian randomization; OR, odds ratio; SNP, single nucleotide polymorphism; UKB, UK Biobank. (TIF) [file pmed.1003967.s022.tif]

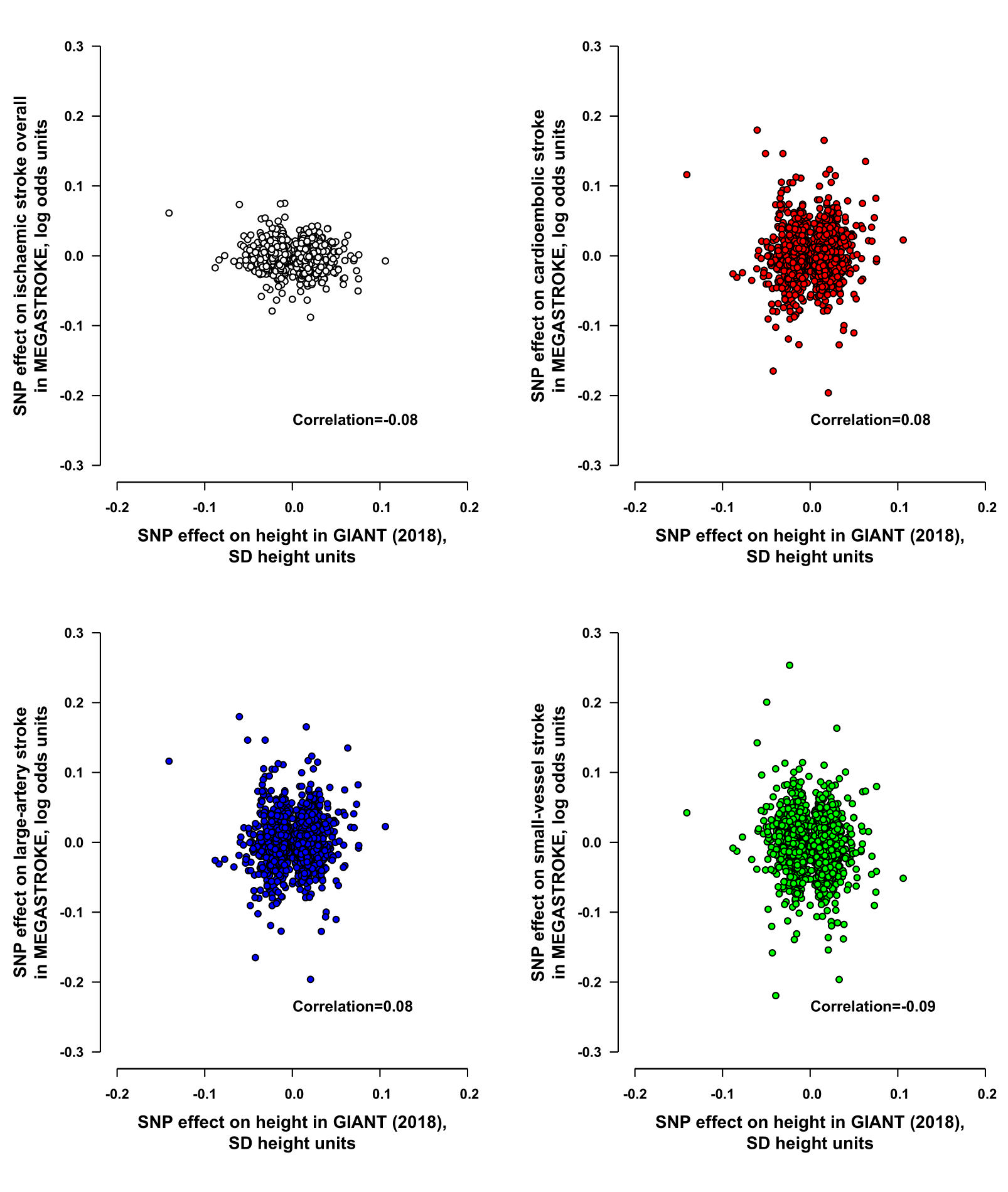

Supplement: S4 Fig — For MEGASTROKE (multiple ancestry), 2,265 height-associated SNPs were available for ischemic stroke cases, 2,270 for cardioembolic and large-artery stroke cases, and 2,084 for small-vessel stroke cases. GIANT (2018), Genetic Investigation of Anthropometric Traits (2018) [18]; SD, standard deviation; SNP, single nucleotide polymorphism;. (TIF) [file pmed.1003967.s023.tif]
